# MYH9-dependent polarization of ATG9B promotes colorectal cancer metastasis by accelerating focal adhesion assembly

**DOI:** 10.1038/s41418-021-00813-z

**Published:** 2021-06-15

**Authors:** Yan Zhong, Ting Long, Chuan-Sha Gu, Jing-Yi Tang, Ling-Fang Gao, Jia-Xian Zhu, Zhi-Yan Hu, Xia Wang, Yi-Dan Ma, Yan-Qing Ding, Zu-Guo Li, Xiao-Yan Wang

**Affiliations:** 1 Department of Pathology, Shenzhen Hospital, Southern Medical University, Shenzhen, Guangdong China; 2 Department of Pathology, School of Basic Medical Sciences, Southern Medical University, Guangzhou, Guangdong China; 3 Key Laboratory of Molecular Tumour Pathology of Guangdong Province, Guangzhou, Guangdong China; 4 Department of Pathology, Nanfang Hospital, Southern Medical University, Guangzhou, Guangdong China

**Keywords:** Metastasis, Oncogenes

## Abstract

Tumour metastasis is a major reason accounting for the poor prognosis of colorectal cancer (CRC), and the discovery of targets in the primary tumours that can predict the risk of CRC metastasis is now urgently needed. In this study, we identified autophagy-related protein 9B (ATG9B) as a key potential target gene for CRC metastasis. High expression of ATG9B in tumour significantly increased the risk of metastasis and poor prognosis of CRC. Mechanistically, we further find that ATG9B promoted CRC invasion mainly through autophagy-independent manner. MYH9 is the pivotal interacting protein for ATG9B functioning, which directly binds to cytoplasmic peptide segments aa368–411 of ATG9B by its head domain. Furthermore, the combination of ATG9B and MYH9 enhance the stability of each other by decreasing their binding to E3 ubiquitin ligase STUB1, therefore preventing them from ubiquitin-mediated degradation, which further amplified the effect of ATG9B and MYH9 in CRC cells. During CRC cell invasion, ATG9B is transported to the cell edge with the assistance of MYH9 and accelerates focal adhesion (FA) assembly through mediating the interaction of endocytosed integrin β1 and Talin-1, which facilitated to integrin β1 activation. Clinically, upregulated expression of ATG9B in human CRC tissue is always accompanied with highly elevated expression of MYH9 and associated with advanced CRC stage and poor prognosis. Taken together, this study highlighted the important role of ATG9B in CRC metastasis by promoting focal adhesion assembly, and ATG9B together with MYH9 can provide a pair of potential therapeutic targets for preventing CRC progression.

## Introduction

Colorectal cancer (CRC) is the third most common malignancy, with around 1.8 million newly diagnosed cases worldwide annually [1]. Forty percent of cases develop metastasis, which is the major reason for the high mortality of CRC [2, 3]. Tumour metastasis is a sophisticated biological process, and the molecular mechanism still cannot be fully explained. Unravelling the molecular mechanisms underlying CRC metastasis is vital to develop early intervention strategies, especially for individuals with high risk of metastasis.

Autophagy is the process to maintain cellular homoeostasis and is regulated by almost 40 autophagy-related proteins (ATGs). To our knowledge, ATGs not only play important roles in autophagy but also contribute prominently to tumour metastasis [4]. Autophagy-related protein 9B (ATG9B) is identified as a multi-spanning membrane protein and participates in autophagosome formation and autophagy initiation in mammalian cells [5]. It has been reported that ATG9B interacts with p38IP and is regulated by p38α mitogen-activated protein kinase pathway [6], which then regulates the trafficking of ATG9B and therefore affects autophagy process in a mammalian system. Moreover, the expression of ATG9B is tissue specific, which is abundant in organs such as placenta and ovary but minimum in testis, liver, lung, muscle, pancreas, and brain, and is important in embryonic development [7]. In our previous study, we identified ATG9B as a downstream target of MALAT-1 [8], which is reported to play an important role in CRC metastasis. To date, few studies on the relationship between ATG9B and tumours have been conducted. Yet the role and mechanism of ATG9B in CRC metastasis remain unclear.

Here we first specifically report that ATG9B is an upregulated protein in CRC and promotes CRC metastasis through enhancing assembly of focal adhesions (FAs) with the assistance of myosin-9 (MYH9). In this context, ATG9B and MYH9 may serve as a pair of potential diagnostic markers and therapeutic targets for CRC metastasis.

## Materials and methods

### Antibodies and small interfering RNA (siRNA) sequences

The antibodies used in the study are listed in Supplementary Table S4, including catalogue numbers, commercial providers and application. siRNA sequences used in the study are listed in Supplementary Table S5.

### Cell lines and cell culture

The CRC cell lines SW480 (CCL-228), SW620 (CCL-227), DLD1 (CCL-221), HCT116 (CCL-247), HT29 (HTB-38), RKO (CRL-2577), LoVo (CCL-229) and HEK293T cells (CRL-3216) were obtained from the American Type Culture Collection (ATCC). These cells were cultured as described previously [9]. We got the cell line certificate of analysis from ATCC. All the cell lines tested negative for mycoplasma.

### Clinical specimens

CRC clinical samples were obtained from patients with pathologically diagnosed with CRC at Nanfang Hospital, Southern Medical University. None of the patients had received any preoperative chemotherapy or radiotherapy. The study was approved by the ethics committee of Nanfang Hospital, Southern Medical University, China.

### Quantitative reverse transcription polymerase chain reaction (qRT-PCR)

Total RNA of CRC cells and CRC tissue were extracted using TRIzol reagent (Takara, 9109). RNA was reverse transcribed to cDNA with a Reverse Transcription Kit (Takara, D6110A). qRT-PCR analysis was performed using SYBR Green Master Mix (Takara, RR420). Primers with the following sequences were used: *ATG9B*, forward 5′-CCC CTC ATA CAA GAA GCT CCC-3′ and reverse 5′-TGC AGG TTG AGC CTG TGT TG-3′; *MYH9*, forward 5′-CCT CAA GGA GCG TTA CTA CTC A-3′ and reverse 5′-CTG TAG GCG GTG TCT GTG AT-3′.

### Immunohistochemistry (IHC)

IHC was performed on paraffin sections of CRC tissue according to standard labelled streptavidin–biotin protocol (Dako) using primary antibodies against ATG9B and MYH9. The degree of staining in the sections was observed and scored independently by two pathologists. The percent positivity of antigen staining was scored from 0 to 4: 0 (0%), 1 (1–25%), 2 (26–50%), 3 (51–75%), and 4 (>75%). The staining intensity was scored on a 4-point scale: 0 (no staining), 1 (weak staining, light yellow), 2 (moderate staining, yellowish brown), and 3 (strong staining, brown). Subsequently, the antigen expression score was calculated by multiplying the percent positivity score and staining intensity score and ranged from 0 to 12. The expression level of antigens was defined as 0 (−), 1–4 (+), 6–8 (++), and 9–12 (+++). Tissue score 0 (−)/1–4(+) were classified into the low-expression group, and tissue scored 6–8 (++)/9–12 (+++) were classified into the high-expression group.

### Construction of lentivirus and stable cell lines

In light of the manufacturer’s instructions, lentiviral constructs containing the indicated *ATG9B*-repressing short hairpin RNA sequence (GAUCCCUGAACAGGAUUAUTT) purchased from Gene Pharma (Suzhou, China) were used to establish cell lines constitutively repressing *ATG9B*. A stable lentiviral vector with overexpression of *ATG9B* (Gene Pharma, Suzhou, China) or *MYH9* (Gene Copoeia, Rockville, USA) were constructed with human full-length cDNA, respectively. CRC cells were transduced with serial dilutions of lentiviral supernatant and selected by using 5 μg/ml puromycin for 2 weeks. The transfection efficiency was confirmed by qRT-PCR and western blots.

### Tumour xenografts

Balb/C-nude mice (female, 4–6 weeks old) were purchased from the Laboratory Animal Centre of Southern Medical University. Mice were housed under pathogen-free conditions in a 12-h dark/light cycle and ad libitum access to food and filtered water. According to our experimental purpose and estimation formula of the sample size, ATG9B tumour metastasis experiments require approximately 48 mice (6 groups). Based on our preliminary experiments, constructing unsuccessful model was about 15-20%. Hence, we took the median censoring rate 17.5% of our previous experiment as our assumed censoring rate,  and estimated 56 as the final number of mice in our experiment. Mice were divided into different groups according to the random number table. For example, 56 mice were involved in ATG9B tumour metastasis experiments, which were evenly divided into 8 groups (7 mice in each group). Firstly, these 56 mice were numbered as 1–56 randomly. Then, 56 mice got a new random number by using random number table. We ordered the random number in ascending order. Then, the order number from 1 to 7 was set as the first group, 8–14 was set as the second group and 15–21 was set as the third group and so on. Accordingly, 49–56 was the last group.The mice with unsuccessful establishment of the tail vein injection were excluded from our study. Finally, we included 48 mice in our data analysis with 8 group (6 mice in each group).

To evaluate the effect of ATG9B on tumour metastasis in vivo, 5 × 10^6^ SW480/ATG9B, DLD1/ATG9B, SW620/shATG9B, LoVo/shATG9B cells and each relevant control cells were injected into nude mice (6 mice per group) via the tail vein. About 60 days later, all mice were sacrificed, lung tissue were collected for metastatic foci evaluation and metastatic tissue were analysed with haematoxylin–eosin staining. All experimental procedures were performed in strict accordance with the recommendations in the Guide for the Care and Use of Laboratory Animals of the National Institutes of Health. The protocol was approved by the Committee on the Ethics of Animal Experiments of Southern Medical University.

### Cell invasion assay

Boyden Transwell chambers (BD Biosciences, 353097) coated with Matrigel (BD Biosciences, 356234) were used following the instructions of the manufacturer. Briefly, 2 × 10^5^ cells were seeded into the upper chambers with Matrigel coated and cultured with serum-free medium. The lower chamber of the transwell was filled with 10% foetal bovine serum culture medium as a chemo-attractant. After 18–24 h incubation, cells that successfully invaded were fixed with 4% paraformaldehyde, stained with 0.1% crystal violet and counted in 5 random visual fields using a light microscope.

### Wound healing assay

Cell migration ability was assessed by the wound healing assay. For the wound healing assay, artificial wounds were scratched on a confluent cell monolayer using sterile tips, and cells were cultured with serum-free medium to prevent proliferation. The cell migration was assessed by measuring the movement of cells into acellular area scratched by a sterile insert. The wound healing images were taken at 0 and 48 h.

### Cell adhesion assay

Twenty four-well plates were coated with fibronectin (FN, BD Biosciences, USA) at 37 °C for 1 h. After blocking with blocking buffer 1% bovine serum albumin, a total of 3 × 10^4^cells were seeded in each well and incubated at 37 °C for 30 min. Then cells were fixed with 4% paraformaldehyde, stained with 0.1% crystal violet and counted in 5 random visual fields using a light microscope.

### Plasmid constructs

*ATG9B*^His^, *STUB1*^Myc^, *Ub*^HA^ plasmids constructed with pcDNA3.1(+) cloning vector were purchased from GENEWIZ company (Guangzhou, China). MYH9^Flag^ plasmid constructed with CMV-M14 cloning vector was purchased from GeneCopia company (Guangzhou, China). Cd1, delpro Cd1 + 2, Cd3 + 4, Cd4, Δ298-332, Δ333-367, Δ368-411, Δ412-438, K374R truncations of His-*ATG9B* and SH3-like, delCCoil, IQCCoil truncations of Flag-*MYH9*, and K48R, K63R truncations of HA-*Ubiquitin* were constructed using PCR.

### His pulldown assays

Bacteria-expressed His or His-tagged ATG9B-Cd2 protein (amino acids 298–438 (aa298–438)) was harvested by His-tag Beaver Beads magnetic beads following the manufacturer’s instructions (Beaver, 70501-5). The purified ATG9B-Cd2^His^ protein was then added to cell lysates of 293T cells transfected with MYH9^Flag^ overnight. The bound proteins were eluted, followed by western blot.

### Immunoprecipitation (IP) and western blot

Cells were lysed in RIPA buffer containing protease inhibitors. Total cell lysates were subjected to IP with the indicated antibodies in protein A/G beads (Santa Cruz Biotechnology, sc-2003). Total protein extracts were resolved on a 10% sodium dodecyl sulfate polyacrylamide gel and electron transferred to polyvinylidene fluoride membranes, which were then blocked in 5% non-fat dry milk in Tris-buffered saline and immunoblotted with primary and secondary antibodies. The protein was detected by ECL chemiluminescence solution (FDbio-Femto, FD8030) and finally visualized using chemiluminescence detection system (Universal Hood II, Bio-Rad). The intensity of the western blot bands was quantified using the NIH ImageJ software.

### Immunofluorescent (IF) staining

Cells grown on confocal dishes were fixed with 4% paraformaldehyde for 30 min and permeabilized with 0.25% Triton X-100 for 10 min at room temperature and stained with the indicated antibodies. Nuclei were visualized with 4,6-diamidino-2-phenylindole. The images were taken with laser scanning confocal microscopy (Zeiss LSM 880 with Airyscan) in Central Laboratory of Southern Medical University.

### Transmission electron microscopy (TEM)

Cells were collected and fixed in 2.5% glutaraldehyde in 100 mM phosphate buffer, then post-fixed in 1% osmic acid for 3 h at 4 °C. They were then embedded in neutral resin, sectioned, doubly stained with uranyl acetate and lead citrate and analysed using TEM (Hitachi, H-7500).

### Nocodazole (NZ) model system to study FA turnover and integrin recycling

This model system is referred to a published study from *Nader*. [10]. In brief, SW480 cells were transiently transfected with ATG9B^EGFP^ plasmid and grown on glass coverslips. After 48 h transfection, cells were treated with 10 μM NZ for 4 h to completely depolymerize microtubes (MTs). Then, NZ was removed and washed out with serum-free medium,  leading to MTs repolymerization followed by FA disassembly. Cells were fixed at appropriate time in 4% paraformaldehyde for 30 min, followed by IF staining. Quantification of integrin β1 and P-Paxillin levels on cell membrane by their mean fluorescent signal intensity was measured by imageJ (Fiji). The plugins of MorphoLibJ in imageJ was used to create cell outline mask. To standardize the  thickness of cell membrane outlined by MorphoLibJ, morphological filters were used and defined the thickness of cell membrane in two pixel radius. A region of interest area was created for a final measure.

### Flow cytometry

Flow cytometry was used to quantify the plasma membrane levels of integrin β1 in the indicated cells. In brief, after centrifugation, integrin β1 antibody was added in cells and determined by fluorescence-activated cell sorter according to the manufacturer’s instruction. Fluorescein isothiocyanate fluorescent intensity was further quantified with the Flow-jo software.

### Statistical analysis

SPSS software for Mac OS version 24.0 was used for statistical analyses. All data followed a normal distribution with homogenous variance. An unpaired two-tailed Student’s *t* test was used to compare two groups with normally distributed data. Pearson’s *χ*^2^ test was applied to analyse the correlation between the expression of ATG9B and clinicopathological features. Kaplan–Meier survival curves were plotted, and log-rank test was performed. Spearman’s correlation analyse was performed to analyse correlated expression levels of ATG9B and MYH9 in CRC tissue. All data are presented as the mean ± standard error of mean (SEM) unless otherwise specified. *P* < 0.05 was considered significant, ***P < 0.001, **P < 0.01, *P < 0.05, NS, no significance.

## Results

### High expression of ATG9B induces metastasis and poor prognosis of CRC

**Fig. 1:**
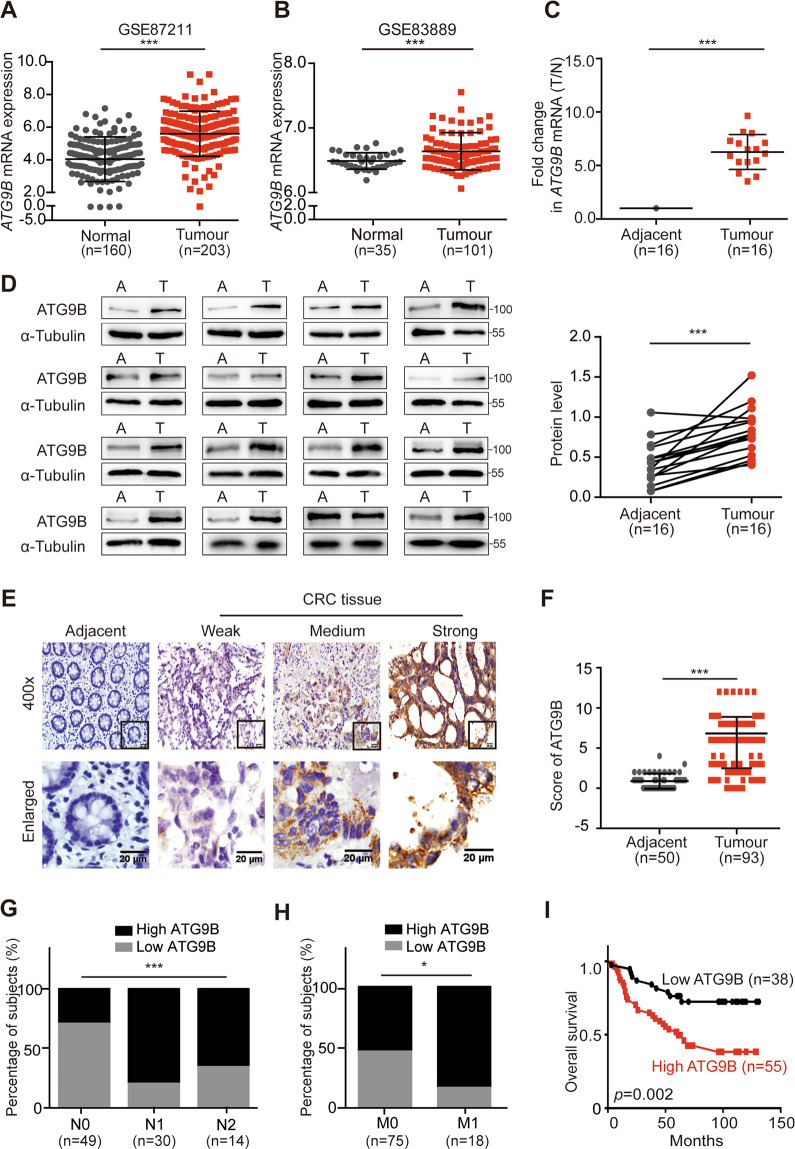
ATG9B is upregulated in CRC tissues and is positively correlated with poor prognosis in CRC patients. **A**, **B** Analysis of ATG9B expression in CRC tissues compared with adjacent normal tissues in the CRC microarray profiles GES87211 (**A**) and GSE83889 (**B**). **C** Fold change (T/N) of *ATG9B* mRNA expression in 16 primary CRC tissues and adjacent normal tissues from the same patient, as determined by RT-PCR. **D** Immunoblot for ATG9B protein expression in 16 human CRC tissues (T) and matched adjacent normal tissues (A) from the same patient. Quantification of protein levels were normalized to those of α-Tubulin are shown in the right panel. **E** Representative ATG9B immunohistochemical staining images of adjacent normal tissue (Adjacent, *n* = 50) and tumour tissue (CRC, *n* = 93) samples (scale bar 20 μm). The magnified parts were displayed in the lower panel. **F** Immunohistochemical score of ATG9B in adjacent tissues and CRC tumour tissues. **G**, **H** Percentage of high and low expression of ATG9B in 93 CRC patients with different N (lymph node metastasis, **G**) and M (distal metastasis, **H**) clinical stages. **I** Kaplan–Meier survival analysis of 93 CRC patients with low and high expression of ATG9B. Data information: Graphs report mean ± SEM. Significance was assessed using two-tailed Student’s *t* test, except for **G**, **H** where Chi-square test was used and **I** where log-rank test was used. ***P < 0.001, **P < 0.01, *P < 0.05.

To investigate the role ATG9B plays in cancer, we analysed the transcriptional datasets assembled from clinical samples in public database ONCOMINE (www.oncomine.org) and discovered that the transcriptional levels of *ATG9B* were dramatically increased in various cancers, especially in CRC (Fig. S1A, B). We further analysed mRNA expression level of ATG9B in the Gene Expression Omnibus (GEO: GSE87211, GSE83889 and GSE32323) database and found that ATG9B expressed significantly higher in CRC tissue than in normal mucosa (Figs. 1A, B and S1C). To confirm this idea, mRNA and protein expression levels of ATG9B in 16 pairs of primary CRC tissue and matched adjacent normal tissue were detected, which showed that a significantly higher expression of ATG9B was found in CRC tissue compared with the matched adjacent normal mucosa (Fig. 1C, D). Subsequently, we detected the expression of ATG9B protein in paraffin-embedded CRC and normal tissue and also found a significantly higher expression of ATG9B in CRC tissue than in normal mucosa (Fig. 1E, F), which was positively associated with high risk of tumour lymph, distal metastasis and advanced Duke’s stage (Fig. 1G, H and Supplementary Table S1). Moreover, high expression of ATG9B was also correlated with metastatic recurrence of CRC (Fig. S1D, E). In addition, Kaplan–Meier survival analysis showed that high expression of ATG9B tightly related to a poorer prognosis of CRC patients (Figs. 1I and S1E). Analysis with univariate and multivariate COX model indicated that the expression level of ATG9B was an independent prognostic factor for patients with CRC (Supplementary Table S2). Taken together, these data demonstrate that ATG9B is positively correlated to tumour metastasis and poor prognosis of CRC patients.

### ATG9B promotes CRC invasion, migration and lung metastasis

**Fig. 2:**
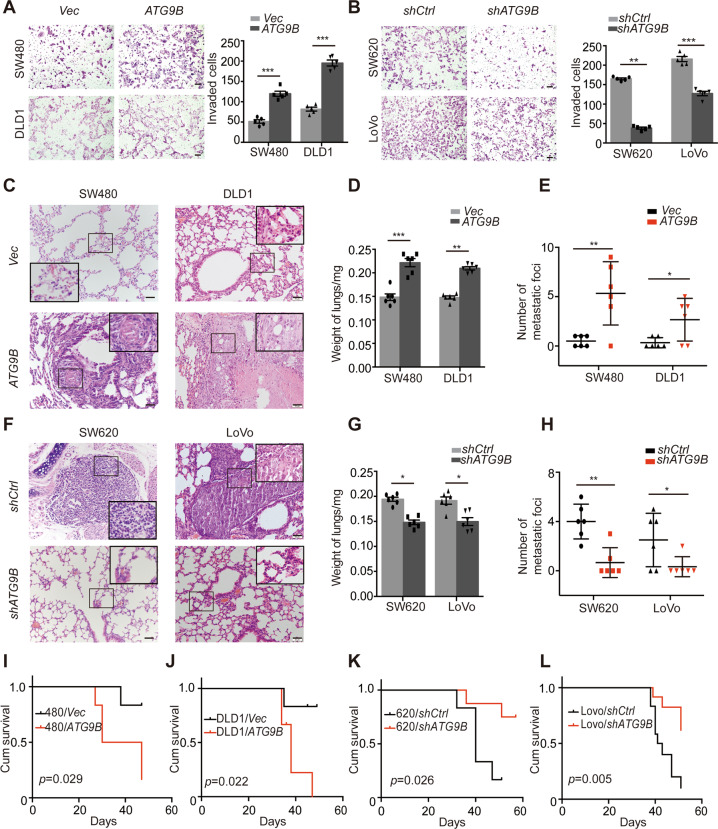
ATG9B promotes CRC cell invasion and lung metastasis. **A**, **B** Boyden chamber invasion assay showing the invasion ability of *ATG9B* upregulation (**A**) and downregulation (**B**) in the indicated cells. Quantification of invaded cells are shown in the right panel (scale bar 50 μm, *n* = 5). **C** Representative H&E staining images of lung metastasis in mice injected with SW480/Vector, SW480/*ATG9B*, DLD1/*Vector* or DLD1/*ATG9B* cells. The black boxes represents the magnified sites (scale bar 20 μm*, n* = 6). **D** Quantification of the lung weights from the experiment like in **C** (*n* = 6). **E** Quantification of metastasis numbers from the experiment like in C (*n* = 6). **F** Representative H&E staining images of lungs metastasis in xenograft-bearing mice with SW620/*shCtrl*, SW620/*shATG9B*, LoVo/*shCtrl* or Lovo/*shATG9B* cells. The black boxes represents the magnified sites (scale bar 20 μm*, n* = 6). **G** Quantification of the lung weights from the experiment like in **F** (*n* = 6). **H** Quantification of metastasis numbers from the experiment like in **F** (*n* = 6). **I**–**L** Kaplan–Meier survival analysis of xenograft-bearing mice with SW480/Ve*ctor* or SW480/*ATG9B* cells (**I**), DLD1/*Vector* or DLD1/*ATG9B* cells (**J**), SW620/*shCtrl* or SW620/*shATG9B* cells (**K**), LoVo/*shCtrl* or Lovo/*shATG9B* cells (**L**). Data information: Graphs report mean ± SEM. Significance was assessed using two-tailed Student’s *t* test, except for **I**–**L** where log-rank test was used. ***P < 0.001, **P < 0.01, *P < 0.05.

To further investigate the role of ATG9B in CRC, *ATG9B*-knockdown SW620 and LoVo cell lines and *ATG9B*-overexpression SW480 and DLD1 cell lines were established (Fig. S2A–E). Next, we conducted transwell invasion assay and wound healing assay which showed that upregulation of ATG9B significantly enhanced cell invasion and migration ability in CRC cells, whereas silencing ATG9B had the opposite effect (Figs. 2A, B and S2F, G). We further applied in vivo tail-vein xenograft models to investigate the role of ATG9B in regulating migration/invasion of CRC cells. The average lung weight and the number of metastatic nodules in the lungs increased in mice inoculated with ATG9B-overexpressing CRC cells (Fig. 2C–E), whereas silencing ATG9B significantly decreased the weight and metastatic nodule number of lungs (Fig. 2F–H). Furthermore, Kaplan–Meier analysis showed that overexpressing ATG9B statistically reduced the overall survival time of mice, but silencing ATG9B prolonged their survival time (Fig. 2I–L). In this context, these data point out that ATG9B is required for the invasion and metastasis of CRC cells.

### ATG9B promotes CRC invasion mainly in an autophagy-independent manner

ATG9B is an important ATG, so we next need to ascertain whether its function in CRC is regulated in an autophagy-dependent manner or not. TEM assay and western blot are presented to show that high expression of ATG9B during starvation could increase the formation of autophagosomes (Fig. S3A), and enhance light chain 3II (LC3II)/I ratio and decrease p62 protein level (Fig. S3B, C), demonstrating that ATG9B can accelerate the process of autophagy during CRC starvation. However, we also found that high expression of ATG9B had no effect on the activation of autophagy in normal culture medium (Fig. S3A–C). Moreover,  interference of key autophagy protein ATG5 or ATG7 in ATG9B-overexpressing cells, the ability of ATG9B on CRC invasion was partially weakened but still obviously stronger than SW480/*Vector* cells (Fig. S3D, E). These results signified that ATG9B’s function in CRC invasion and metastasis is mainly autophagy independent.

### Direct interaction between ATG9B and MYH9

**Fig. 3:**
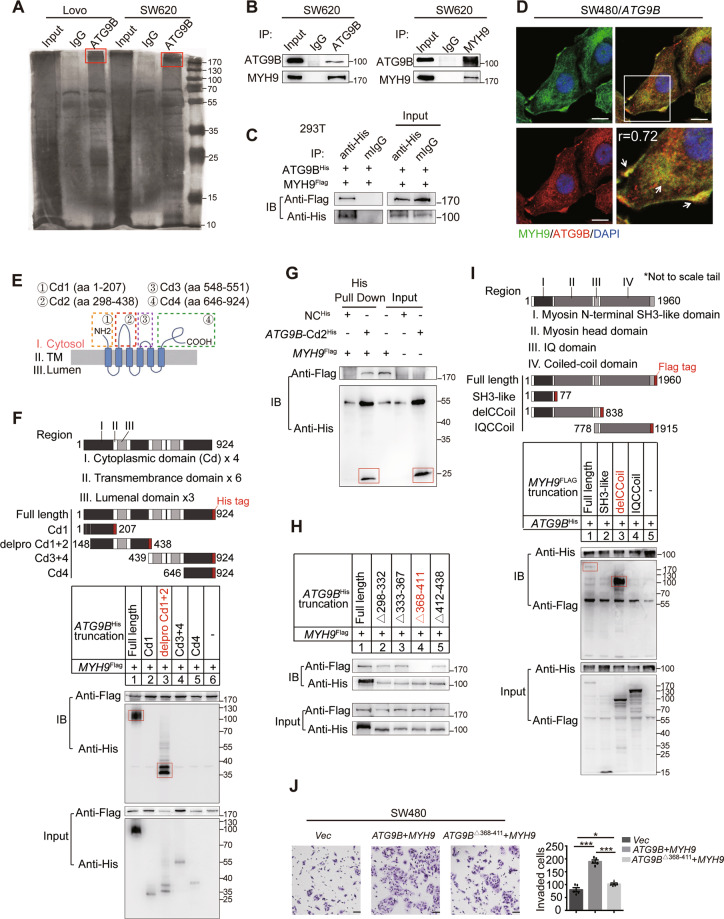
MYH9 interacts with ATG9B directly. **A** LoVo and SW620 cells were lysed and subjected to immunoprecipitation with anti-ATG9B antibody or rabbit IgG using silver staining. The red boxes represents the detected bands. **B** Endogenous ATG9B and MYH9 were immunoprecipitated in SW620 cells. **C** Exogenous ATG9B^His^ and MYH9^Flag^ were immunoprecipitated in 293T cells. **D** ATG9B colocalized with MYH9 in SW480/ATG9B cells. The magnified parts are displayed in the lower right panel and the arrows points to the colocalized sites (scale bar 10 μm, *r* (Person’s correlation coefficient) = 0.72). **E** Cytoplasmic segments of ATG9B, including aa1–207 (Cd1), aa298–438 (Cd2), aa548–551 (Cd3) and aa646–924 (Cd4). **F** Diagrammatic representation of ATG9B and its truncated forms. Based on sequence and structure analyses, region I (cytoplasmic domain), region II (transmembrane domain) and region III (luminal domain) are indicated. 293T cells were transfected with the indicated constructs subjected to immunoprecipitation with anti-Flag (against MYH9). Immunoblot analysis was performed with anti-Flag or anti-His (against ATG9B). The red boxes represents the pull-down bands. **G** Direct binding of *MYH9*^Flag^ to *ATG9B*-Cd2^His^ using His pulldown assay. The red boxes represents the pull-down bands. **H** Constructs with deleted mutations Δ298-332, Δ333-367, Δ368-411 and Δ412-438 of *ATG9B-*Cd2^His^ were used for immunoprecipitation with MYH9^Flag^. **I** Diagrammatic representation of MYH9 and its truncated forms. 293T cells were transfected with the indicated constructs subjected to immunoprecipitation with anti-His (against ATG9B). The red boxes represents the pull-down bands. **J** Transwell invasion assay detected SW480 cells transfected with vector, *ATG9B*^His^ together with *MYH9*^Flag^ or *ATG9B*^Δ368-411^ together with *MYH9*^Flag^ (scale bar 50 μm, *n* = 5). Data information: Graphs report mean ± SEM. Significance was assessed using two-tailed Student’s *t* test. ***P < 0.001, **P < 0.01, *P < 0.05.

To identify the underlying molecules of ATG9B involved in promoting CRC invasion and metastasis, IP assay was applied. A differential band at approximately >170 KD was analysed by liquid chromatography–mass spectrometry (LC-MS) (Fig. 3A). COG function classification analysis demonstrated that the proteins found above were involved in various biological activities, including posttranslational modification, protein turnover, chaperones and cytoskeleton rearrangement (Fig. S4A), among which MYH9 had the highest score (Fig. S4B, C). Then the interactions between endogenously expressed ATG9B and MYH9 were confirmed in SW620 and LoVo cells (Figs. 3B and S4D), and the interactions between exogenously expressed His-tagged ATG9B (*ATG9B*^His^) and Flag-tagged MYH9 (*MYH9*^Flag^) were confirmed in HEK293T cells (Fig. 3C). Moreover, IF assay validated a significant co-localization of ATG9B and MYH9 in the cytoplasm and the cell edge of CRC (Fig. 3D). These findings strongly suggested that ATG9B interacts with MYH9 in CRC cells.

ATG9B is predicted to be a multi-transmembrane protein anchored in intracellular vesicles, with its N-terminus and C-terminus in the cytoplasmic facet, by the online TMHMM website (www.cbs.dtu.dk/services/TMHMM/). Since MYH9 is a cytoplasmic myosin protein, binding may occur only on the cytoplasmic surface of ATG9B, so we constructed four truncations of *ATG9B* containing four different cytoplasmic domains (Cds; Fig. 3E). Then, four *ATG9B* truncations fused with His-tag together with *MYH9* cDNAs were transfected into HEK293T cells. By co-immunoprecipitation (Co-IP) experiments, MYH9 co-immunoprecipitated with the second Cd (Cd2) of ATG9B (Fig. 3F). Additionally, to investigate whether ATG9B binds to MYH9 directly, Cd2 domain of human ATG9B protein was purified with IDA-Nickel His beads. His pull-down assay showed that MYH9 directly bound to *ATG9B*-Cd2^His^ but not His control (Fig. 3G). To further ascertain the accurate binding site of ATG9B, four deleted mutations of *ATG9B*-Cd2^His^ were constructed. Co-IP showed that aa368–411 of ATG9B was necessary for the direct interaction between ATG9B and MYH9 (Fig. 3H).

Furthermore, to investigate which domain of MYH9 is crucial for ATG9B binding, three MYH9^Flag^ truncations were constructed and the result of Co-IP assay indicated that ATG9B co-immunoprecipitated with the head domain (aa77–777) of MYH9 (Fig. 3I). I-TASSER and Phyre2 were adopted to mimic the structures of ATG9B and MYH9. ZDOCK was used to build an interaction model of these two proteins (Fig. S4E). Interestingly, we found that P726, G732, F733 and K731 of MYH9 might play a key role in interacting with ATG9B-Cd2 (Fig. S4F). In addition, MYH9 together with WT-*ATG9B*, but not *ATG9B*^Δ368-411^, prominently promoted the invasion of SW480 cells (Fig. 3J). Taken together, these data demonstrate that ATG9B regulates invasion of CRC cells by interacting with MYH9 directly.

### ATG9B and MYH9 enhance the protein stability of each other

**Fig. 4:**
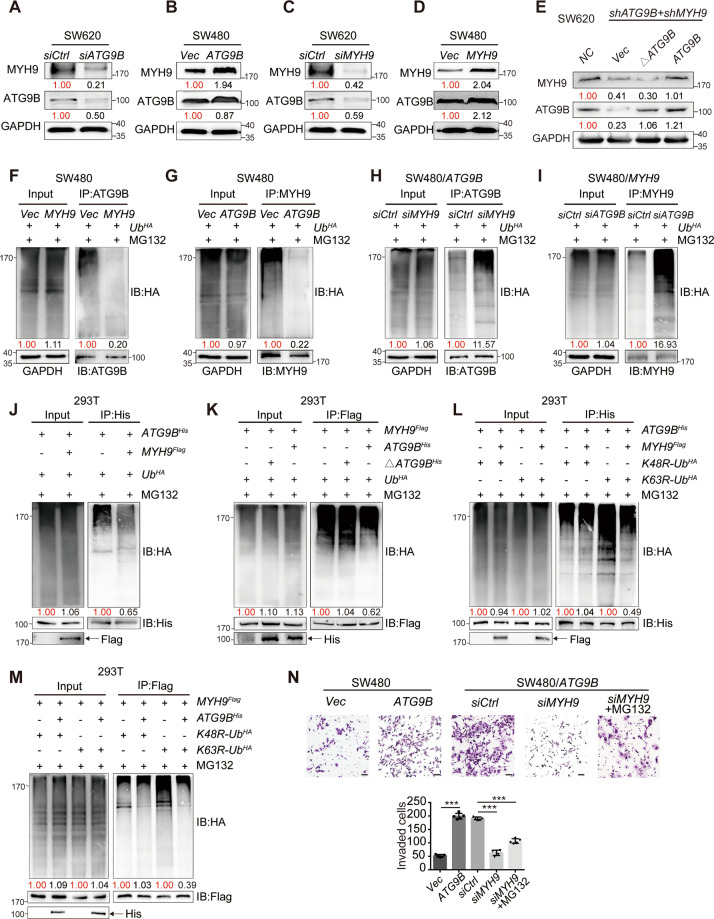
ATG9B and MYH9 reciprocally reduce protein ubiquitination level. **A**, **B** Western blot shows the MYH9 protein expression levels in SW620/*siCtrl* and SW620/*siATG9B* cells (**A**) or SW480/*Vector* and SW480/*ATG9B* cells (**B**). **C**, **D** Western blot shows the ATG9B protein expression levels in SW620/*siCtrl* and SW620/*siMYH9* (**C**) or SW480/*Vector* and SW480/*MYH9* cells (**D**). **E** Western blot shows the protein expression level of MYH9 in SW620/*shATG9B* + *shMYH9* cells transfected with WT-*ATG9B* or *ATG9B*^Δ368-411^ plasmids. **F** Co-IP shows that HA-ubiquitin is pulled down by ATG9B in SW480/*Vector* or SW480/*MYH9* cells. **G** Co-IP shows that HA-ubiquitin is pulled down by MYH9 in SW480/*vector* or SW480/*ATG9B* cells. **H** Co-IP shows that HA-ubiquitin is pulled down by endogenous ATG9B in SW480/*ATG9B* cells with control or MYH9 knockdown. **I** Co-IP shows that HA-ubiquitin is pulled down by endogenous MYH9 in SW480/*MYH9* cells with control or *ATG9B* knockdown. **J** Co-IP analysis using 293T cells transfected with the indicated plasmids and treated with MG132. **K** Co-IP analysis using 293T cells transfected with plasmids encoding *Ub*^HA^, *MYH9*^Flag^, or *ATG9B*^His^, or Δ*ATG9B*^His^ (deleted mutation with aa368–411 of ATG9B) and treated with MG132. **L** Co-IP analysis using 293T cells transfected with plasmids encoding *ATG9B*^His^, together with *MYH9*^Flag^ or *HA-K48R-Ubiquitin* (*K48R-Ub*^HA^) and *HA-K63R-Ubiquitin* (K63R-Ub^HA^) and treated with MG132 to detect His-*ATG9B* ubiquitination. **M** Co-IP analysis using 293T cells transfected with the indicated plasmids and treated with MG132 to detect Flag-*MYH9* ubiquitination. **N** Transwell invasion assay detected SW480/*Vector*, SW480/*ATG9B* cells and SW480/*ATG9B* cells transfected with siRNA-control (*siCtrl*), *siMYH9* or *siMYH9* treated with MG132. Quantification of invaded cells are shown in the right panel (scale bar 50 μm, *n* = 5). Data information: Graphs report mean ± SEM, *n* = 3. Quantification of average protein levels or ubiquitination levels were normalized to those of GAPDH or IB protein listed under the bands, and each *Control/Vector* group was normalized as 1.00 marked in red, and the value of the experimental group was compared with it marked in black. Significance was assessed using two-tailed Student’s *t* test. ***P < 0.001.

Next, we found that ATG9B and MYH9 have mutual positive regulation at the protein level but not at mRNA levels (Figs. 4A–D and S5A–F). Furthermore, overexpression of full length but not the deletion of aa368–411 of ATG9B rescued MYH9 protein expression in SW620 cells with ATG9B and MYH9 knockdown (Fig. 4E). It indicated that the interaction is in a protein-dependent manner, which was initiated during posttranslational stage. In support of this notion, protein stability of ATG9B and MYH9 was examined. As shown in Fig. S5G, H, knockdown of ATG9B in CRC cells substantially decreased the half-life of MYH9 protein, while ectopic expression of ATG9B increased the half-life of MYH9 protein. A similar change in the degradation rate of ATG9B could be observed when upregulating or downregulating MYH9 expression (Fig. S6I, J). Therefore, these findings suggested a mutual protein–protein stabilization between ATG9B and MYH9.

### The stabilization of ATG9B–MYH9 complex impairs K48 ubiquitinated degradation

The autophagy–lysosome and ubiquitin–proteasome pathways are the two main routes for protein degradation [11]. To further determine which pathway is responsible for ATG9B and MYH9 turnover, SW480/*ATG9B* and SW480/*MYH9* cells were treated with chloroquine (CQ, a lysosome inhibitor) or MG132 (a proteasome inhibitor). Results showed that the reduction of ATG9B caused by MYH9 knockdown, as well as the reduction of MYH9 caused by ATG9B knockdown, was strongly rescued after MG132 treatment (Fig. S5K, L). However, no significant change was found after CQ treatment (Fig. S5M, N). Furthermore, elevated MYH9 or ATG9B levels resulted in a significant reduction of ATG9B or MYH9 ubiquitination, respectively (Fig. 4F, G), whereas silencing MYH9 or ATG9B induced the ubiquitin accumulation of each other (Fig. 4H, I). We further found that ATG9B ubiquitination was markedly reduced by overexpressing MYH9 (Fig. 4J), and the ubiquitination level of MYH9 was only reduced by wild-type *ATG9B*^His^, but not *ATG9B*^His^ with aa368–411 mutation (Fig. 4K). Furthermore, to detect what type of ubiquitination was related to the interaction of ATG9B and MYH9, we constructed HA*-Ubiquitin*-K48R mutant (K48 lysine mutated to arginine) and HA-*Ubiquitin*-K63R mutant (K63 lysine is mutated to arginine) to perform Co-IP assay. Results showed that K63R mediated ubiquitination of ATG9B still could be reduced by MYH9, while MYH9 cannot decrease the ubiquitination of ATG9B mediated by K48R-Ubiquitin (Fig. 4L). We also found the similar result in Flag-MYH9 Co-IP (Fig. 4M). It indicated that K48 of ubiquitin plays an important role in mediating ATG9B-MYH9 ubiquitination. These findings strongly suggested that the inhibition of K48 ubiquitinated degradation was the primary reason accounting for the mutual stabilization of ATG9B and MYH9.

Functionally, ATG9B-overexpressing cells were highly invasive, whereas silencing of MYH9 significantly suppressed the invasion of SW480/ATG9B cells. Simultaneously, adding MG132 to inhibit the degradation of ATG9B caused by the reduction of MYH9 will restore a part of invasion, but it is still weaker than the control group, indicating that ATG9B depends on MYH9 to promote CRC invasion (Fig. 4N).

### The interaction of ATG9B and MYH9 inhibits STUB1-mediated ubiquitination

**Fig. 5:**
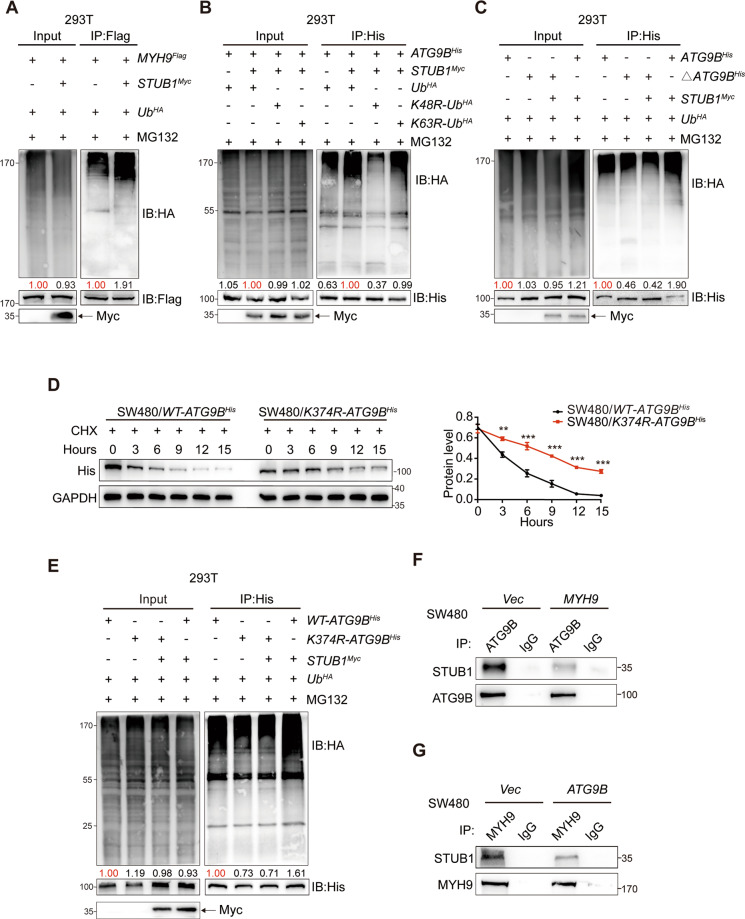
ATG9B and MYH9 interaction inhibits STUB1-mediated ubiquitination. **A** Co-IP analysis using 293T cells transfected with the indicated plasmids and treated with MG132. **B** Co-IP analysis using 293T cells transfected with the indicated plasmids and treated with MG132 to detect His-ATG9B ubiquitination. **C** Co-IP analysis using 293T cells transfected with plasmids encoding *Ub*^HA^, *STUB1*^Myc^, *ATG9B*^His^ or Δ*ATG9B*^His^ (deleted mutation with aa368–411 of ATG9B) and treated with MG132. **D** The degradation protein level of His detected in SW480/*WT-ATG9B*^His^ and SW480/*K374R-ATG9B*^His^ cells with CHX treatment (50 μg/ml). **E** Co-IP analysis using 293T cells transfected with the indicated plasmids and treated with MG132 to detect His-ATG9B ubiquitination. **F** The immunoprecipitation of STUB1 and ATG9B was detected in SW480 cells transfected with *Vector* or *MYH9* plasmids. **G** The immunoprecipitation of STUB1 and MYH9 was detected in SW480 cells transfected with Vector or ATG9B plasmids. Data information: Graphs report mean ± SEM. Quantification of ubiquitination levels were normalized to those of IB protein listed under the bands, and each *Control/Vector* group was normalized as 1.00 marked in red, and the value of the experimental group was compared with it marked in black. Significance was assessed using two-tailed Student’s *t* test. ***P < 0.001, **P < 0.01, *P < 0.05.

Since E3 ubiquitin ligases are required for the transfer of ubiquitin to the substrates [12], we supposed that the decreased ubiquitination level of ATG9B and MYH9 resulted from the inhibition of E3 ubiquitin ligases binding to the ATG9B–MYH9 complex. The carboxyl terminus of hsc70-interacting protein (CHIP, also known as STUB1), was the most potential ubiquitin ligases of ATG9B predicted from UbiBrowser website (http://ubibrowser.ncpsb.org/ubibrowser/; Fig. S6A). Co-IP assay revealed that STUB1 indeed interacted with ATG9B and MYH9 (Fig. S6B). Ectopic expression of STUB1 significantly led to the reduction of MYH9 and ATG9B in CRC cells (Fig. S6C). It suggested that STUB1 can negatively regulate ATG9B and MYH9 protein expression. Next, we verified that STUB1 can promote the ubiquitination of MYH9 (Fig. 5A) and ATG9B (Fig. 5B) and further ascertained that STUB1 participates in ATG9B K48 ubiquitination (Fig. 5B). Moreover, His-Co-IP assay confirmed that aa368–411 of ATG9B was the vital fragment for STUB1-mediated ubiquitination (Fig. 5C).

There was a unique lysine residue (K374) in aa368–411 of ATG9B, suggesting that K374 of ATG9B might be the key ubiquitination sites of STUB1 (Fig. S6D). Indeed, K374 mutation (K374R) can prolong the half-life of ATG9B (Fig. 5D). Compared with WT-ATG9B^His^, the amount of HA-ubiquitin immunoprecipitated by His was reduced when K374 was mutated, and STUB1 cannot enhance the ubiquitination level of K374R-ATG9B^His^. It demonstrated that K374 is a vital site for STUB1-mediated ATG9B ubiquitination (Fig. 5E). Moreover, we also found that K374 mutation of ATG9B still can interact with MYH9 (Fig. S6E) and does not enhance invasive ability of ATG9B in CRC, compared with WT-ATG9B (Fig. S6F), indicating that the function of K374R-ATG9B is as same as WT-ATG9B because K374 mutation might not affect ATG9B–MYH9 interaction. Importantly, although STUB1 was an E3 ubiquitin ligase of ATG9B and MYH9, ATG9B and MYH9 would preferentially interact with each other rather than with STUB1 (Fig. 5F, G). These findings suggested that aa368–411 of ATG9B is not only the fragment for MYH9 direct interaction but also has the ubiquitination site K374 for STUB1 binding.

### ATG9B is carried to the cell edge by MYH9 to accelerate FA assembly

**Fig. 6:**
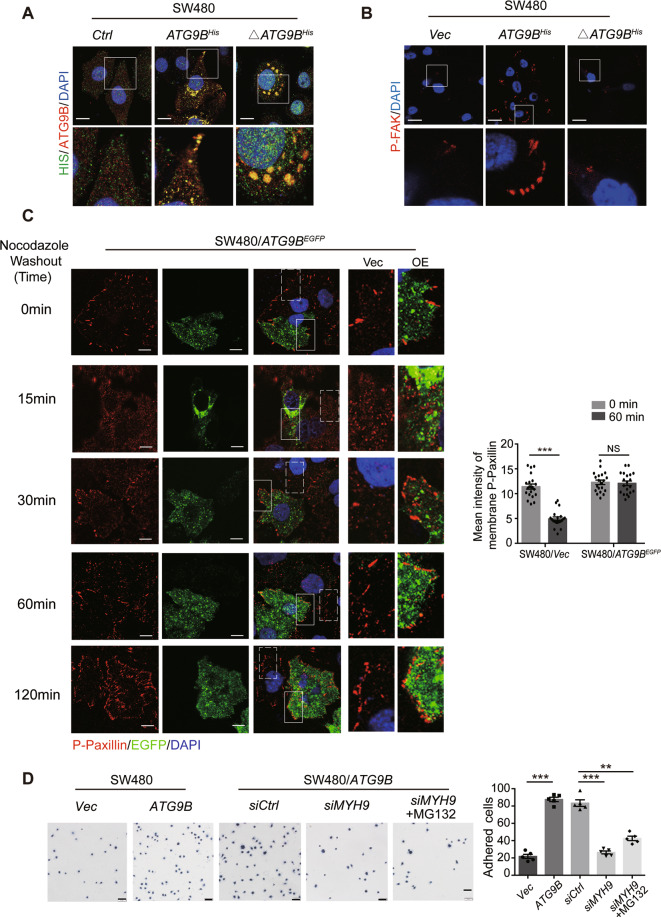
ATG9B is trafficking to the cell edge through MYH9 to promote the focal adhesion assembly. **A** Double IF staining detected the distribution of exogenous ATG9B (yellow colour) in SW480 cells transfected with *vector*, *ATG9B*^His^ or Δ*ATG9B*^His^ (deleted mutation with aa368–411 of ATG9B). The magnified parts are displayed in lower panel, scale bar 10 μm. **B** IF staining to detect pY397-FAK (red) expression in the indicated cells. The magnified parts are displayed in lower panel, scale bar 10 μm. **C** Immunofluorescence images of pY118-Paxiilin in SW480/*ATG9B*^EGFP^ cells after treatment with 10 μM nocodazole (NZ) for 4 h followed by washout for the indicated times, scale bar 10 μm. The magnified parts of vector cells (dotted line) and *ATG9B*^EGFP^ overexpression cells (solid line) were, respectively, displayed. Mean intensity levels of surface pY397-Paxillin at the indicated times of NZ washout is presented in the right panel (*n* = 20 cells analysed per group). **D** Time-limited FN adhesion assay to detect the rate of FA formation in the indicated cells for 30 min. Scale bar 50 μm (*n* = 5). Data information: Graphs report mean ± SEM. Significance was assessed using two-tailed Student’s *t* test. ***P < 0.001, **P < 0.01, NS, no significance.

IF assay showed overexpressing ATG9B made itself to be transported from peri-nuclear area to cell leading edge, and such transportation almost disappeared in the absence of MYH9, even using MG132 to inhibit ATG9B degradation (Fig. S7A, B). Additional double staining of His and ATG9B indicated that ATG9B^Δ368-411^ could not be carried to the cell edge without MYH9 participation (Figs. 6A and S7C), signifying that ATG9B is transported from peri-nuclear to cell edge in a MYH9-dependent manner. Kyoto Encyclopedia of Genes and Genomes enrichment analysis of the proteins pulled down by ATG9B in LC-MS identified that ATG9B can positively enrich FA pathway, which propagated signals through integrins reassembling FAs from peri-nuclear area to the cell edge (Fig. S7D). Thus, further investigation was applied to find out the role of ATG9B in the formation of FAs. IF showed that biochemical markers of FA assembly, pY397-FAK and pY118-Paxillin were remarkably increased at the cell edge in ATG9B-overexpressing cells. However, silencing MYH9 dramatically eliminated the effect of ATG9B on FA formation, even after the treatment of MG132 (Fig. S7E). We also found the same results in immunoblot assay (Fig. S7F). Moreover, we found that overexpressing ATG9B^Δ368-411^ had no effect on FA assembly of CRC cells (Fig. 6B), indicating that ATG9B promotes FA assembly relied on MYH9 interaction. To further visually detect the function of ATG9B in FA assembly, we conducted NZ model system, which was used to study FA turnover [10]. NZ can promote depolymerization of MTs in cells, and the polymerization of MTs directly targets FA disassembly [13]. Therefore, after NZ was washed out, MTs were repolymerized and FAs were following disassembled. Next, the reassembly of FAs happened and facilitated to observe. Results showed that, at 15 min after NZ washout, pY118-Paxillin was scattered expressed in cytoplasm in cells whether transfected ATG9B^EGFP^ or not. Interestingly, pY118-Paxillin reformation at membrane was observed in ATG9B^EGFP^ cells but not in control cells at 30–60 min after NZ washout in the same field of view (Fig. 6C). These results suggested that ATG9B can accelerate FA assembly in CRC cells. Furthermore, overexpressing ATG9B enhanced adhesion of tumour cells to FN, while silencing MYH9 could reverse this phenomenon, even in the presence of MG132 (Fig. 6D). These findings strongly indicated that ATG9B played an important role in accelerating FA assembly by being transported to the cell edge in a MYH9-dependent manner.

### ATG9B initiates FA assembly by mediating integrin β1 activation and polarization

**Fig. 7:**
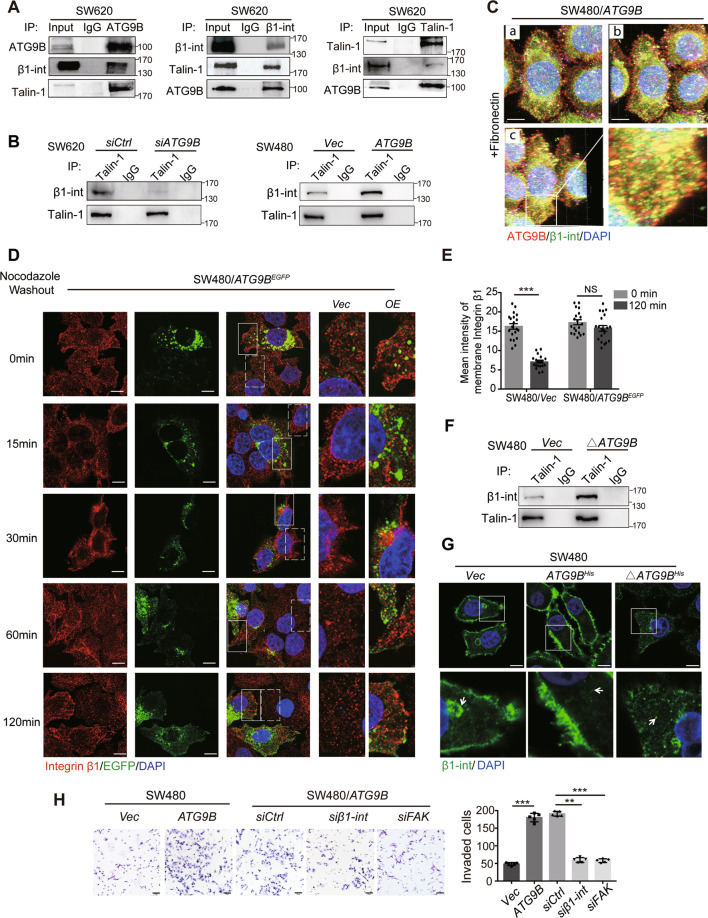
ATG9B participates in FA formation through mediating integrin β1 activation and polarization. **A** Co-IP assays were applied to detect the immunoprecipitation of ATG9B, integrin β1 and Talin-1 in SW620 cells. **B** The immunoprecipitation of Talin-1 and integrin β1 was detected in SW620 cells transfected with *si-Control* or *si-ATG9B* (left panel) and in SW480 cells transfected with vector or *ATG9B* plasmids (right panel). **C** Three-dimensional shooting presented the colocalization of ATG9B and integrin β1 in fibronectin-coated dishes. a Frontage shooting (0°). b Shooting of rotating 45° to the right. c Shooting of rotating 225° to the right. The magnified parts are displayed in the lower right panel, scale bar 10 μm. **D** Immunofluorescence images of integrin β1 in SW480/ATG9B^EGFP^ cells after treatment with 10 μM nocodazole (NZ) for 4 h followed by washout for the indicated times, scale bar 10 μm. The magnified parts of *vector* cells (dotted line) and *ATG9B*^EGFP^ overexpression cells (solid line) were respectively displayed in the right panel. **E** Mean intensity levels of surface integrin β1 at the indicated times of NZ washout is presented in the right panel (*n* = 20 cells analysed per group). **F** The immunoprecipitation of Talin-1 and integrin β1 was detected in SW480 cells transfected with vector and Δ*ATG9B* (deleted mutation with aa368–411 of ATG9B) plasmids. **G** The distribution of integrin β1 detected by IF staining in the indicated cells. The magnified parts are displayed in the lower panel, scale bar 10 μm. The arrows represents the intracellular localization. **H** Transwell invasion assay was used to analyse the invasive ability of the indicated cells. Quantification of invaded cells are shown in the right panel (scale bar 50 μm, *n* = 5). Data information: Graphs report mean ± SEM. Significance was assessed using two-tailed Student’s *t* test. ***P < 0.001, **P < 0.01, NS, no significance.

It has been well defined that the activation of integrin β1 is the trigger for FA formation [13, 14]. Talin-1 functions as the canonical activator for integrin β1 by binding to its cytoplasmic tails [15, 16], which was pulled down by ATG9B proved by LC-MS (Fig. S4B). Accordingly, to explore whether ATG9B mediated Talin-1/integrin β1 to participate in FA formation, Co-IP assay primarily confirmed the interaction of ATG9B, Talin-1 and integrin β1 (Fig. 7A). Notably, silencing or overexpression of ATG9B positively affected the binding of Talin-1 with integrin β1 (Fig. 7B). It suggested that ATG9B might play an indispensable role in interaction between Talin-1 and integrin β1, which in turn promotes the activation of integrin β1.

After activation in cytoplasm, endocytosed integrin requires continuously being transported to cell leading edge for reassembling new FAs [16]. Two- (Fig. S8A) and three-dimensional (Fig. 7C and Supplementary Movie S1 for three-dimensional shooting) IF assay showed that much dot-like co-localization signalling of ATG9B and integrin β1 occurred not only at the cell edge but also in the cytoplasm. NZ model system found that, at 15–30 min after NZ washout, integrin β1 was endocytosed and distributed in peri-nuclear area in cells whether transfected ATG9B^EGFP^ or not. Intriguingly, integrin β1 was recycled back to the cell edge observed in ATG9B^EGFP^ cells but not in control cells at 60 min after NZ washout in the same field of view (Fig. 7D, E). These results suggested that ATG9B can accelerate integrin β1 polarization to the membrane to reassemble FAs in CRC cells. Moreover, we found that ATG9B^Δ368-411^, which was deleted at the interaction sites with MYH9, can also tight up the binding of integrin β1 and Talin-1 (Fig. 7F), but could not increase integrin β1 clustered at the cell edge (Fig. 7G). Flow cytometric assay also confirmed that overexpression of ATG9B can enhance fluorescence intensity of membrane integrin β1. However, silencing MYH9 in SW480/*ATG9B* cells decreased integrin β1 intensity, which could not be rescued after MG132 treatment (Fig. S8B, C). These results suggested that ATG9B took part in integrin polarization relied on MYH9. Further invasive assay showed that silencing integrin β1 or FA kinase dramatically reduced ATG9B-dependent cell invasion (Fig. 7H), indicating that ATG9B promoted the invasion of CRC by participating in FA formation. In summary, these findings strongly suggested that ATG9B is not only a key factor for integrin β1 activation but also an intrinsic basis for integrin β1 polarization to the cell edge for FA formation.

### Simultaneously high expression of ATG9B and MYH9 acts as prognosis of CRC patients

**Fig. 8:**
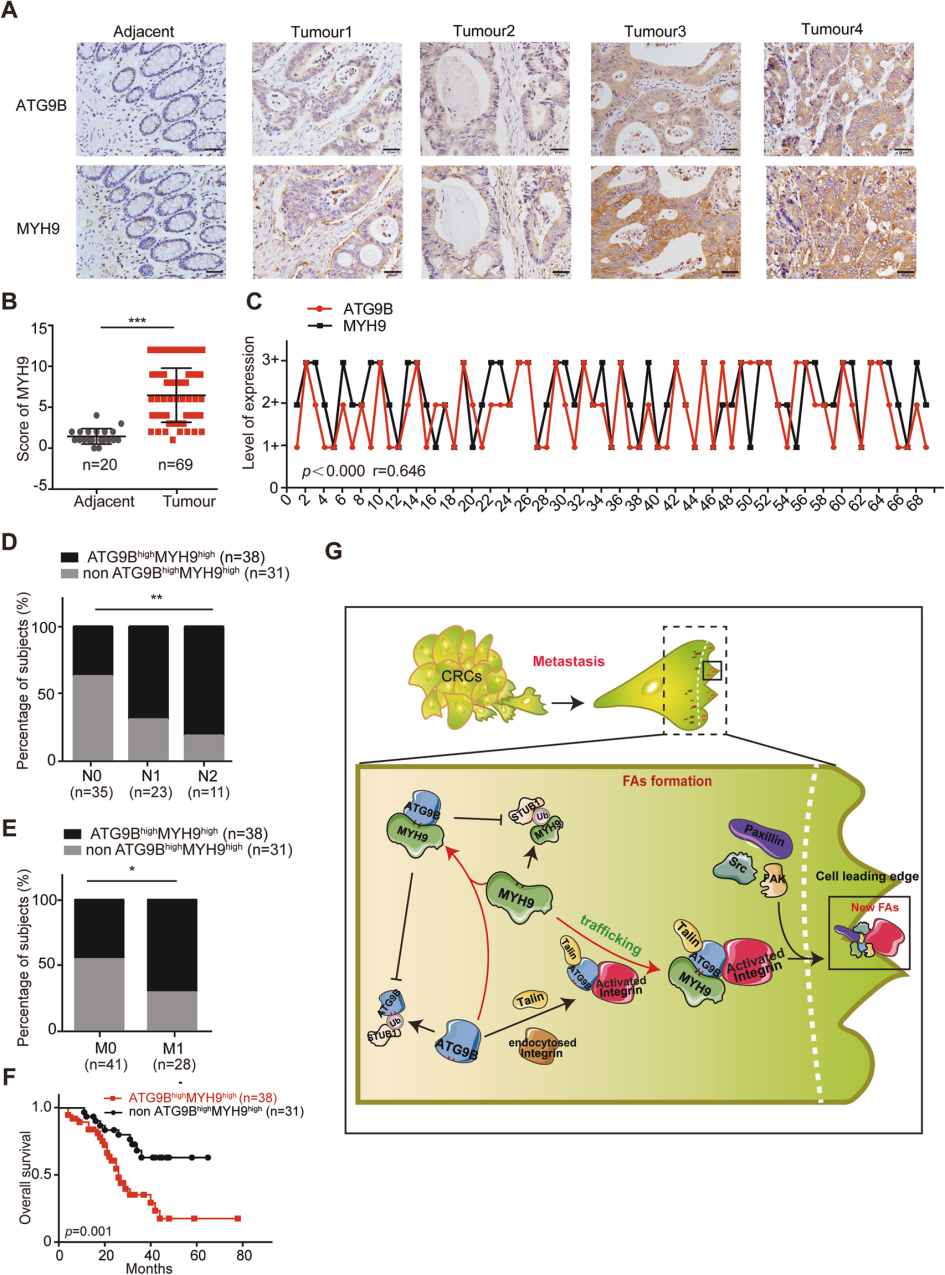
ATG9B correlates with MYH9 expression in CRC and concurrent expression is indicative of patient survival. **A** Representative ATG9B and MYH9 immunohistochemical staining images in serial sections from the same adjacent normal tissue (Adjacent, *n* = 20) and tumour tissue (CRC, *n* = 69) samples, scale bar 20 μm. **B** Immunohistochemical score of MYH9 in 20 adjacent normal tissue and 69 CRC tissue. **C** Spearman’s correlation analyses between ATG9B and MYH9 expressions in CRC tissues. The level of 1+ represented for the score of 1–4, and 2+ represented for the score of 6–8, and 3+ represented for the score of 9–12. Spearman *r* = 0.646, *n* = 69. **D**, **E** Percentage of ATG9B^high^ MYH9^high^ (simultaneously high expression of ATG9B and MYH9) or non-ATG9B^high^ MYH9^high^ (non-high co-expression of ATG9B and MYH9) in 69 patients with different N (lymph node metastasis, **D**) and M (distal metastasis, **E**) clinical stages. **F** Kaplan–Meier survival analysis of 69 CRC patients with ATG9B^high^ MYH9^high^ (simultaneously high expression of ATG9B and MYH9) and non-ATG9B^high^ MYH9^high^ (non-high co-expression of ATG9B and MYH9) according to our data. **G** The mechanism of ATG9B promotes CRC metastasis through accelerating FA assembly. The white dashed line are displayed as the boundaries. The right side of the line represented for the area of the cell leading edge and the left side of the line represented for the area of intracellular. ATG9B increased the formation of FAs by promoting endocytosed integrin β1 activation, which was continuously polarized to the cell edge by MYH9-dependent transportation. Moreover, ATG9B directly interacted with MYH9, which blocked their ubiquitination sites and therefore prevented them from degradation, thus continuously activated their function in CRC cells. Data information: Graphs report mean ± SEM. Significances were assessed using two-tailed Student’s *t* test (**B**), Spearman’s correlation test (**C**), Chi-square test (**D**, **E**) and log-rank test (**F**). ***P < 0.001, **P < 0.01, *P < 0.05.

Clinically, we found that the protein expression of MYH9 increased in paraffin-embedded CRC tissue (Fig. 8A, B). Furthermore, Spearman’s correlation analyses revealed positive correlation between ATG9B and MYH9 expression levels in serial sections from the same CRC patients (Fig. 8C). Simultaneously high expression of ATG9B and MYH9 was closely associated with high risk of tumour lymph node invasion and distant metastasis (Fig. 8D, E and Supplementary Table S3). Importantly, Kaplan–Meier survival analysis revealed that patients with both high expression of ATG9B and MYH9 (ATG9B^high^MYH9^high^) was related to poorer prognosis compared with the non-ATG9B^high^MYH9^high^ group, which comprised of ATG9B^low^MYH9^high^, ATG9B^high^MYH9^low^ and ATG9B^low^MYH9^low^ (Fig. 8F). All in all, these results highlighted that simultaneously high expression of ATG9B and MYH9 could predict a high rate of metastatic recurrence and poor survival of CRC patients.

## Discussion

Our previous research revealed that long non-coding RNA *MALAT-1* was highly expressed in CRC and closely related to metastasis [9]. Furthermore, we screened at least 243 potential genes that associated with CRC metastasis through *MALAT-1*-related genome-wide expression profiling analysis, including *ATG9B* [8]. However, to date, very few detailed studies have been performed to delineate the complex relationship between ATG9B and tumour progression. In this study, we discovered that ATG9B expression is positively correlated with metastasis, advanced stage, and poor survival in human CRC patients. In vitro and in vivo studies confirmed that ATG9B enhanced the migration/invasion property of CRC cells by interacting with MYH9. ATG9B and MYH9 positively regulate each other at the protein level and synergistically enhance protein stability. The combination of ATG9B and MYH9 decreased the binding of STUB1 to them and reduced each other’s proteasomal degradation. Further investigation revealed that ATG9B increased the formation of FAs by promoting the activation of integrin β1, which was subsequently transported to the front edge of cell in a MYH9-denpendent manner. Taken together, we highlighted the vital role of ATG9B in tumour metastasis by accelerating the formation of FAs in a MYH9-dependent manner during cell migration (Fig. 8G).

*ATG9* is identified as the only transmembrane protein among autophagy-related genes that can be detected in different membrane compartments, including small vesicles that reside near the Golgi, mitochondria and the PAS [17]. It has been well defined that *ATG9* has two different orthologues, *ATG9A* and *ATG9B* [18]. Recent studies reported that ATG9B is highly expressed in some cancer, including cervical cancer [19] and renal clear cell carcinoma [20], while it is lower expressed in hepatocellular carcinoma [21] and breast cancer [22]. However, in breast cancer, study showed that downregulation of ATG9B but not its orthologue ATG9A could accelerate breast cancer progression [22], signifying that ATG9A and ATG9B have different biological function in cancer. Here we first reported the correlation between high expression of ATG9B and CRC development and further clarified the function and mechanism underlying this process. Furthermore, we suggested that ATG9B could be a valid biomarker for predicting the risk of metastasis and poor prognosis of CRC patients.

Cells cultured in normal medium have extremely low autophagic activity. The induction of autophagy in cells requires starvation treatment (using Earle’s balance salt solutions) or endoplasmic reticulum stress (using Brefeldin A) or mammalian target of rapamycin pathway inhibition (using Rapamycin) [23]. We first found that ATG9B can only regulate autophagy process during starvation in CRC cells, which cannot affect autophagy in well-nourished environment. In this research, we did not use any treatments to induce autophagy and surprisingly found that, as an ATG, ATG9B also can promote CRC progression, suggesting that the function of ATG9B in CRC is not mainly dependent on autophagy activation.

Subsequently, we identified the protein MYH9 as an interacting protein of ATG9B. MYH9, also called non-muscle myosin IIA (NMM-IIA), is encoded by *MYH9* and has been characterized as regulating various cellular responses, including cell invasion, cell polarity and migration [24, 25]. Further study confirmed that the cytoplasmic peptide segments aa368–411 of ATG9B indeed directly binds to the head domain of MYH9. Moreover, overexpressing ATG9B promoted the protein expression of MYH9 but not significant change was found in its transcription level, suggesting that ATG9B participated in the posttranslational modification of MYH9. Surprisingly, we confirmed that MYH9 also has the same effect on ATG9B. Furthermore, the combination prevented the binding of STUB1, indicating that the ATG9B and MYH9 interaction may cause a structural change for each other, and resulted in the reduction of ubiquitination and degradation, thus maintaining high protein expression level of ATG9B and MYH9 in CRC cells. In tumours, there may also be two or more  proteins, which belong to different families, could combine with each other and is similar to the binding manner of ATG9B and MYH9, thus contributing to mutual stabilization of proteins promoting cascade amplification of tumour pathways. Tumour metastasis depends on the dynamic regulation of cell adhesion through integrin β1 [26]. During FA formation, integrin is continuously internalized and subsequently participates in endosomal recycling, which polarized to the cell leading edge to drive adhesion dynamics [10]. In the process of integrin activation, Talin-1 functions as a key trigger for maintaining endocytosed integrins in an active conformation [27]. The binding of Talin-1 and integrin β1 subsequently changes the tilt angle of transmembrane domain and Cds of integrin, therefore shifting integrin from the conformation of ‘bent-closed’ to the ‘extended-open’ [27]. However, the endocytosed integrins was encapsulated in endosomes, while Talin-1 was distributed in the cytoplasm outside the endosomes. Whether there is an intermediary in helping the connection of endocytosed integrins and Talin-1 still remains unknown. In this context, ATG9B inlayed in endosomes may act as a medium mediating the binding of endocytosed integrin and cytoplasmic Talin-1, therefore facilitating integrin β1 activation. In addition, we further found that MYH9 is the vital trafficking factor for ATG9B/integrin β1 polarization. Overexpression of ATG9B accelerated integrin’s membranization through the assistance of MYH9. Such evidence demonstrated that ATG9B not only acts as a mediator to promote integrin β1 activation but also provides a connection to hang integrin compartment to MYH9, which is then carried to the cell edge for accelerating FA assembly.

It has been reported that ATG1/ULK1 acts as an upstream signal for actomyosin activation and ATG9A trafficking during the process of autophagy in *Drosophila* cells and *Human* breast cancer cells [28]. However, we preliminarily confirmed that ULK1 had the opposite function on ATG9B and MYH9, and ULK1 may not be their upstream signal in CRC invasion (Fig. S9A–C). We are still urgently searching for the trigger for ATG9B–MYH9 trafficking in CRC cells.

In conclusion, our studies demonstrated that ATG9B can promote the invasion and metastasis of CRC by accelerating the formation of FAs in a MYH9-dependent manner. To the best of our knowledge, no previous studies have addressed the distinct role of ATG9B in CRC and the mutual regulation of ATG9B and MYH9. This study suggests targeting interaction between ATG9B and MYH9 may be a promising therapeutic strategy for CRC patients.

## Supplementary information

Supplementary Figure legends

Supplementary Figure 1

Supplementary Figure 2

Supplementary Figure 3

Supplementary Figure 4

Supplementary Figure 5

Supplementary Figure 6

Supplementary Figure 7

Supplementary Figure 8

Supplementary Figure 9

Supplementary Table S1

Supplementary Table S2

Supplementary Table S3

Supplementary Table S4

Supplementary Table S5

Supplementary Movie S1

## References

[1] Siegel RL, Miller KD, Fedewa SA, Ahnen DJ, Meester RGS, Barzi A (2017). Colorectal cancer statistics, 2017. CA Cancer J Clin.

[2] Ferlay J, Soerjomataram I, Dikshit R, Eser S, Mathers C, Rebelo M (2015). Cancer incidence and mortality worldwide: sources, methods and major patterns in GLOBOCAN 2012. Int J Cancer.

[3] Libanje F, Raingeaud J, Luan R, Thomas Z, Zajac O, Veiga J (2019). ROCK2 inhibition triggers the collective invasion of colorectal adenocarcinomas. EMBO J.

[4] Katheder NS, Khezri R, O’Farrell F, Schultz SW, Jain A, Rahman MM (2017). Microenvironmental autophagy promotes tumour growth. Nature..

[5] Chan EY, Longatti A, McKnight NC, Tooze SA (2009). Kinase-inactivated ULK proteins inhibit autophagy via their conserved C-terminal domains using an Atg13-independent mechanism. Mol Cell Biol.

[6] Webber JL, Tooze SA (2010). Coordinated regulation of autophagy by p38alpha MAPK through mAtg9 and p38IP. EMBO J.

[7] Ungermann C, Reggiori F. Atg9 proteins, not so different after all. Autophagy. 2018;14:1456–9. 10.1080/15548627.2018.1477382 PMC6103728 29966469

[8] Yang MH, Hu ZY, Xu C, Xie LY, Wang XY, Chen SY (2015). MALAT1 promotes colorectal cancer cell proliferation/migration/invasion via PRKA kinase anchor protein 9. Biochim Biophys Acta.

[9] Hu ZY, Wang XY, Guo WB, Xie LY, Huang YQ, Liu YP (2016). Long non-coding RNA MALAT1 increases AKAP-9 expression by promoting SRPK1-catalyzed SRSF1 phosphorylation in colorectal cancer cells. Oncotarget..

[10] Nader GP, Ezratty EJ, Gundersen GG (2016). FAK, talin and PIPKI*γ* regulate endocytosed integrin activation to polarize focal adhesion assembly. Nat Cell Biol.

[11] Ciechanover A (2005). Proteolysis: from the lysosome to ubiquitin and the proteasome. Nat Rev Mol Cell Biol.

[12] Zhao CX, Zeng CM, Wang K, He QJ, Yang B, Zhou FF, et al. Ubiquitin-proteasome system-targeted therapy for uveal melanoma: what is the evidence? Acta Pharmacol Sin. 2021;42:179–88. 10.1038/s41401-020-0441-3 PMC8027653 32601365

[13] Ezratty EJ, Partridge MA, Gundersen GG (2005). Microtubule-induced focal adhesion disassembly is mediated by dynamin and focal adhesion kinase. Nat Cell Biol.

[14] Mygind KJ, Schwarz J, Sahgal P, Ivaska J, Kveiborg M. Loss of ADAM9 expression impairs β1 integrin endocytosis, focal adhesion formation and cancer cell migration. J Cell Sci. 2018;131:jcs205393. 10.1242/jcs.205393 29142101

[15] Malinin NL, Pluskota E, Byzova TV (2012). Integrin signaling in vascular function. Curr Opin Hematol..

[16] Chorev DS, Moscovitz O, Geiger B, Sharon M (2014). Regulation of focal adhesion formation by a vinculin-Arp2/3 hybrid complex. Nat Commun..

[17] Yamamoto H, Kakuta S, Watanabe TM, Kitamura A, Sekito T, Kondo-Kakuta C (2012). Atg9 vesicles are an important membrane source during early steps of autophagosome formation. J Cell Biol..

[18] Militello RD, Colombo MI (2011). A membrane is born: origin of the autophagosomal compartment. Curr Mol Med..

[19] Tingting C, Shizhou Y, Songfa Z, Junfen X, Weiguo L, Xiaodong C (2019). Human papillomavirus 16E6/E7 activates autophagy via Atg9B and LAMP1 in cervical cancer cells. Cancer Med..

[20] Ma Z, Qi Z, Shan Z, Li J, Yang J, Xu Z. The role of CRP and ATG9B expression in clear cell renal cell carcinoma. Biosci Rep. 2017;37:BSR20171082. 10.1042/BSR20171082 PMC5686392 28923830

[21] Wang N, Tan HY, Li S, Feng Y (2017). Atg9b deficiency suppresses autophagy and potentiates endoplasmic reticulum stress-associated hepatocyte apoptosis in hepatocarcinogenesis. Theranostics..

[22] Zhang X, Li C, Wang D, Chen Q, Li CL, Li HJ (2016). Aberrant methylation of ATG2B, ATG4D, ATG9A and ATG9B CpG island promoter is associated with decreased mRNA expression in sporadic breast carcinoma. Gene..

[23] Klionsky DJ, Abdel-Aziz AK, Abdelfatah S, Abdellatif M, Abdoli A, Abel S, et al. Guidelines for the use and interpretation of assays for monitoring autophagy (4th edition). Autophagy. 2021;17:1–382. 10.1080/15548627.2020.1797280 PMC7996087 33634751

[24] Li F, Shi J, Xu Z, Yao X, Mou T, Yu J (2018). S100A4-MYH9 axis promote migration and invasion of gastric cancer cells by inducing TGF-beta-mediated epithelial-mesenchymal transition. J Cancer..

[25] Liao Q, Li R, Zhou R, Pan Z, Xu L, Ding Y (2017). LIM kinase 1 interacts with myosin-9 and alpha-actinin-4 and promotes colorectal cancer progression. Br J Cancer..

[26] Samarelli AV, Ziegler T, Meves A, Fässler R, Böttcher RT. Rabgap1 promotes recycling of active β1 integrins to support effective cell migration. J Cell Sci. 2020;133:jcs243683. 10.1242/jcs.243683 PMC7522031 32843574

[27] Hamidi H, Ivaska J. Every step of the way: integrins in cancer progression and metastasis. Nat Rev Cancer. 2018;18:533–48. 10.1038/s41568-018-0038-z PMC6629548 30002479

[28] Tang HW, Wang YB, Wang SL, Wu MH, Lin SY, Chen GC (2011). Atg1-mediated myosin II activation regulates autophagosome formation during starvation-induced autophagy. EMBO J..

