# Supplementary material for: MYH9-dependent polarization of ATG9B promotes colorectal cancer metastasis by accelerating focal adhesion assembly
Source: Cell Death Differ. 2021 Jun 15;28(12):3251–69. doi: 10.1038/s41418-021-00813-z (PMC8629984; doi:10.1038/s41418-021-00813-z)
Supplement: Supplementary file 1 — Supplementary Figure legends [file 41418_2021_813_MOESM1_ESM.docx]

**Supplementary Figure legends**

**Supplementary Figure S1. (Related to Fig 1): Expression and prognosis of *ATG9B* in public databases.**

**A** Outlier profile of *ATG9B* expression in the Oncomine database. The numbers in the red and green boxes in the Outlier column of the table denoted the number of datasets that showed upregulation and downregulation of *ATG9B*, respectively. The right panel represents the COPA statistics of *ATG9B* in 17 CRC datasets. **B** Analysis of *ATG9B* expression in different kinds of CRC tissue compared with normal colon tissue in the CRC microarray profiles Kaiser Colon. **C** Analysis of *ATG9B* expression in CRC tissues compared with adjacent normal tissues in the CRC microarray profiles GSE32323. **D** High mRNA expression level of *ATG9B* was related to metastatic recurrence of CRC in CRC microarray profiles GSE21510. **E** Statistical analysis of *ATG9B* mRNA expression in CRC specimens with metastasis classification in the Tsukamoto Colorectal dataset. **F** High mRNA expression level of *ATG9B* is correlated with poor survival of CRC patients in GSE39582.

**Data information:** Graphs report mean ± SEM. Significance was assessed using 2-tailed Student’s *t*-test, except for (F) where log-rank test was used. ****P*< 0.001, **P*< 0.05.

**Supplementary Figure S2.** **(Related to Fig 2): ATG9B promotes the migration of CRC cells.**

**A, B** ATG9B expression in a panel of CRC cell lines at the mRNA level (A) and the protein level (B). **C, D** The expression of *ATG9B* were detected by qRT-PCR (C) and western blot (D) in ATG9B-knockdown SW620 and control cells. **E** Expression of ATG9B in CRC cells were detected by western blot with ATG9B-overexpression or -knockdown. **F, G** Wound healing assays was performed to detect SW480 and DLD1 cells with *ATG9B* upregulation (F, *ATG9B* versus vector) and SW620 and LoVo cells with *ATG9B* downregulation (G, Sh*ATG9B* versus *shCtrl*), scale bar 50 μm, *n* = 5.

**Data information:** Graphs report mean ± SEM. Quantification of average protein levels were normalized to those of GAPDH under the bands, and each *Control/Vector* group was normalized as 1.00 marked in red, and the value of the experimental group was compared with it marked in black. Significance was assessed using 2-tailed Student’s *t*-test, ****P*< 0.001, ***P*< 0.01, **P*< 0.05.

**Supplementary Figure S3: The function of ATG9B in CRC is not mainly through autophagy pathway.**

**A** The formation of autophagosomes was detected using transmission electron microscopy in SW480/vector and SW480/ATG9B cells in the condition of normal culture medium (Med) and EBSS treatment (scale bar 2 μm in upper panel, scale bar 500 nm in lower panel, *n* = 10). **B** Western blotting was performed to detect the process of autophagy affected by ATG9B in the condition of normal culture medium (Med) and EBSS treatment. Qualification of LC3 in using lower strips (LC3 II) divided by upper strips (LC3 I) to get the LC3 II/I ratio in the right panel. **C** Western blotting was performed to detect the expression of indicated proteins when ATG5 or ATG7 knockdown in SW480/ATG9B cells. **D** Boyden chamber invasion assay shows the invasion ability of indicated cells, *n = 5.*

**Data information:** Graphs report mean ± SEM. Quantification of average protein levels were normalized to those of GAPDH under the bands (except LC3II/I was described above), and each *Control/Vector* group was normalized as 1.00 marked in red, and the value of the experimental group was compared with it marked in black. Significance was assessed using 2-tailed Student’s *t*-test. *** *P* < 0.001, ** *P* < 0.01, * *P* < 0.05, NS, no significance.

**Supplementary Figure S4. (Related to Fig 3): The direct interaction between ATG9B and MYH9.**

**A** COG function classification analysis of ATG9B pull-down proteins. **B** Top5 of ATG9B pull-down proteins detected by LC-MS analysis. **C** Mass spectrum of a representative peptide fragment from the protein band corresponding to MYH9. **D** ATG9B and MYH9 interaction was determined by CoIP analyses in LoVo cells. **E** I-TASSER and Phyre2 displayed the mimic structure of ATG9B-Cd2 (aa298-438) and MYH9-delCCoil (aa1-838) and molecular mimic docking of this two fragments (right panel). The red box represented for the binding sites of ATG9B-Cd2 and MYH9-delCCoil. **F** ZDOCK used to predict the binding sites of ATG9B and MYH9.

# Supplementary Figure S5. (Related to Fig 4): ATG9B and MYH9 can enhance each other’s protein stability.

**A, B** The expression of *MYH9* were detected by qRT-PCR (A) and western blot (B) in SW480/*ATG9B-siMYH9* and control cells (*siCtrl*). **C, D** The mRNA expression levels of *MYH9* in SW620/*siCtrl* and SW620/*siATG9B* cells (C) or SW480/*Vector* and SW480/*ATG9B* cells (D). **E, F** The mRNA expression levels of *ATG9B* in SW620/*siCtrl* and SW620/*siMYH9* (E) or SW480/*Vector* and SW480/*MYH9* cells (F)*.* **G, H** The degradation protein level of MYH9 detected in SW620/*siCtrl* and SW620/*siATG9B* cells (G) or SW480/*Vector* and SW480/*ATG9B* cells (H) with CHX treatment (50 μg/ml). **I, J** The degradation protein level of ATG9B detected in SW620/*siCtrl* and SW620/*siMYH9* (I) or SW480/*Vector* and SW480/*MYH9* cells (J) with CHX treatment (50 μg/ml). **K** Western blot shows ATG9B protein expression levels in SW480/*ATG9B* cells after *MYH9* knockdown and treated with MG132 (25 μM for 8 h). **L** Western blot shows MYH9 expression in SW480/*MYH9* cells after *ATG9B* knockdown and treated with MG132. **M** Western blot was performed to detect ATG9B protein expression levels in SW480/*ATG9B* cells after MYH9 knockdown and treated with CQ (50 μM for 4h). **N** Western blot was performed to detect MYH9 expression in SW480/*MYH9* cells after *ATG9B* knockdown and treated with CQ.

**Data information:** Graphs report mean ± SEM, *n* = 3. Quantification of average protein levels were normalized to those of GAPDH under the bands, and each *Control/Vector* group was normalized as 1.00 marked in red, and the value of the experimental group was compared with it marked in black. Significance was assessed using 2-tailed Student’s *t*-test. ****P*< 0.001, ***P*< 0.01, **P*< 0.05, NS, no significance.

**Supplementary Figure S6.** **(Related to Fig 5): STUB1 can mediated ATG9B K374 ubiquitination.**

**A** The probable ubiquitin ligases of ATG9B predicted from UbiBrowser website (<http://ubibrowser.ncpsb.org/)>. **B** Endogenous STUB1, ATG9B and MYH9 were immunoprecipitated in SW620 cells. **C** Protein levels of ATG9B and MYH9 in SW620 cells transfected with vector or STUB1. Quantification of average protein levels were normalized to those of GAPDH listed under the bands, and *Vector* group was normalized as 1.00 marked in red, and the value of the experimental group was compared with it marked in black. **D** The unique lysine residue (K374) of ATG9B fragment (aa368-aa411). **E** Exogenous *K374R-ATG9B*^His^ and *MYH9*^Flag^ were immunoprecipitated in 293T cells. **F** Boyden chamber invasion assay showing the invasion ability of *WT-ATG9B*^His^ and *K374R-ATG9B*^His^ up-regulation in SW480 cells. Quantification of invaded cells were shown in the lower panel (scale bar 50 μm, *n* = 5).

**Data information:** Graphs report mean ± SEM, Significance was assessed using 2-tailed Student’s *t*-test, NS, no significance.

**Supplementary Figure S7. (Related to Fig 6): ATG9B activated the expression of key focal adhesion proteins through MYH9.**

**A, B** The distribution of ATG9B (red, upper penal) and MYH9 (red, lower panel) were observed by IF staining in indicated cells, scale bar 10 μm. **C** Western blot shows protein expression of ATG9B, His and MYH9 in SW480 cells transfected with vector, ATG9B^His^ or △ATG9B^His^ (deleted mutation aa368-411 of ATG9B) plasmids. Quantification of average protein levels were normalized to those of GAPDH listed under the bands (*n* = 3). **D** The focal adhesion pathway was enriched by ATG9B. The proteins in red frames were the interactive proteins with ATG9B detected by LC-MS. **E** IF staining was applied to observe the expression of pY118-paxillin (green, upper panels) or pY397-FAK (green, lower pannels) together with cytoskeletal actin (red) in indicated cells. The magnified parts were displayed in the lower panel of each target, scale bar 10 μm. **F** Western blot analysis was performed to measure the expression of ATG9B, MYH9 and the main members of the focal adhesion signaling pathway in the indicated cells.

**Data information:** Quantification of average protein levels were normalized to those of GAPDH listed under the bands, and each *Control/Vector* group was normalized as 1.00 marked in red, and the value of the experimental group was compared with it marked in black.

**Supplementary Figure S8. (Related to Fig 7): ATG9B promotes membrane expression of Integrin β1.**

**A** ATG9B colocalized with integrin β1 in SW480/*ATG9B* cells in PBS (upper panel) or in fibronectin (lower panel, 2.5 μg/cm^2^) coated dishes. The magnified parts were displayed in the rightmost, scale bar 10 μm. The arrows represented for the colocalized spots. **B, C** FACS detection of Integrin β1 membrane expression on indicated cells. *n* = 3.

**Data information:** Graphs report mean ± SEM, Significance was assessed using 2-tailed Student’s *t*-test, **P*< 0.05.

**Supplementary Figure S9: The functional role of ULK1 in CRC.**

**A** Boyden chamber invasion assay shows the invasion ability of Vector and ULK1 up-regulation in SW480 cells. Quantification of invaded cells were shown in the right panel (scale bar 50 μm, *n* = 5). **B** Wound healing assays for SW480 cells with Vector and ULK1 up-regulation (scale bar 100 μm, *n* = 3). **C** Western blot was performed to detect the indicated protein mediated by ULK1 overexpression. Quantification of average protein levels were normalized to GAPDH listed under the bands, and *Vector* group was normalized as 1.00 marked in red, and the value of the experimental group was compared with it marked in black.

**Data information:** Graphs report mean ± SEM. Significance was assessed using 2-tailed Student’s *t*-test. And (D) compared to control group at each corresponding time point. *** *P* < 0.001.
