# Supplementary figures and images for: MYH9-dependent polarization of ATG9B promotes colorectal cancer metastasis by accelerating focal adhesion assembly
Source: Cell Death Differ. 2021 Jun 15;28(12):3251–69. doi: 10.1038/s41418-021-00813-z (PMC8629984; doi:10.1038/s41418-021-00813-z)

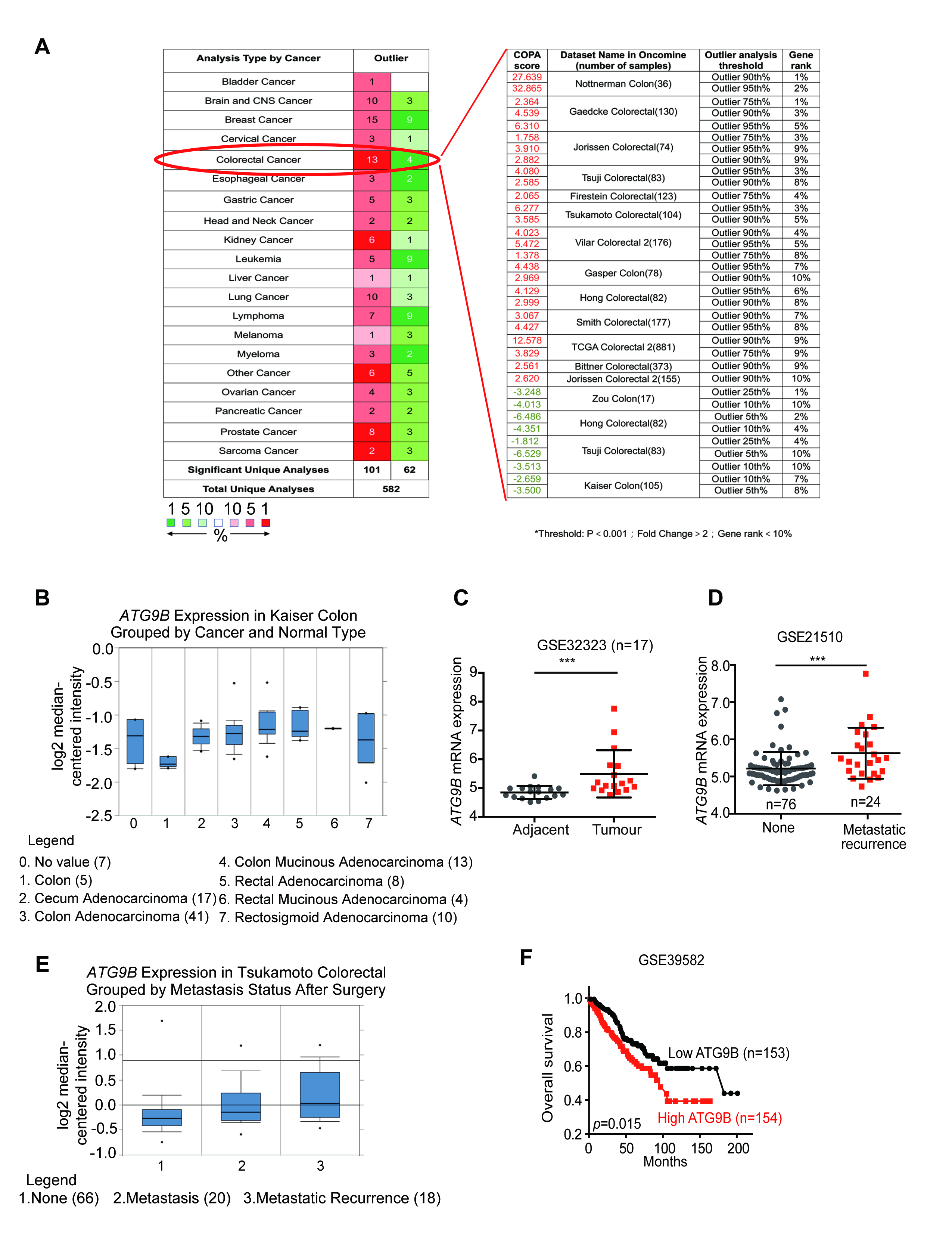

Supplement: Supplementary file 2 — Supplementary Figure 1 [file 41418_2021_813_MOESM2_ESM.tif]

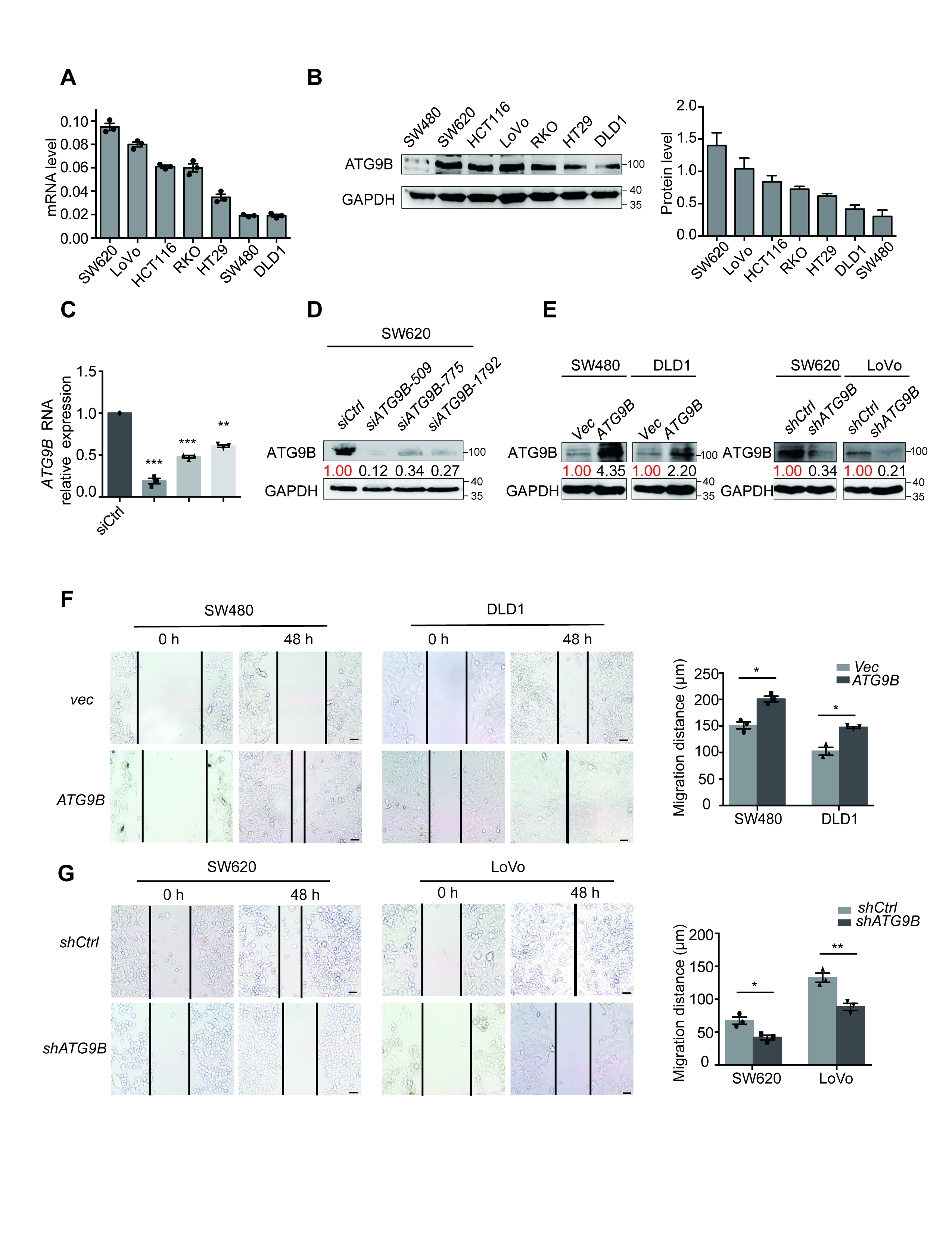

Supplement: Supplementary file 3 — Supplementary Figure 2 [file 41418_2021_813_MOESM3_ESM.tif]

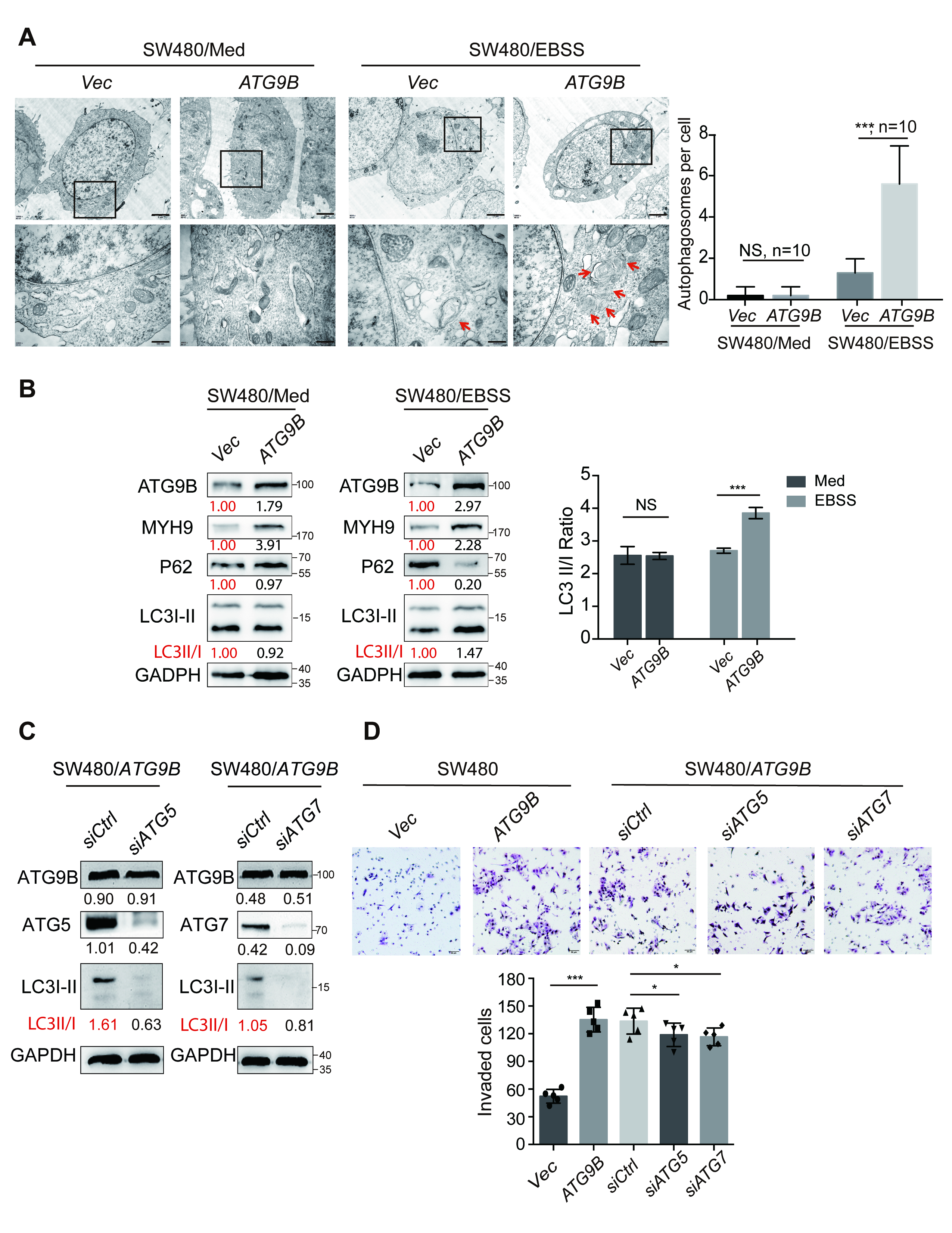

Supplement: Supplementary file 4 — Supplementary Figure 3 [file 41418_2021_813_MOESM4_ESM.tif]

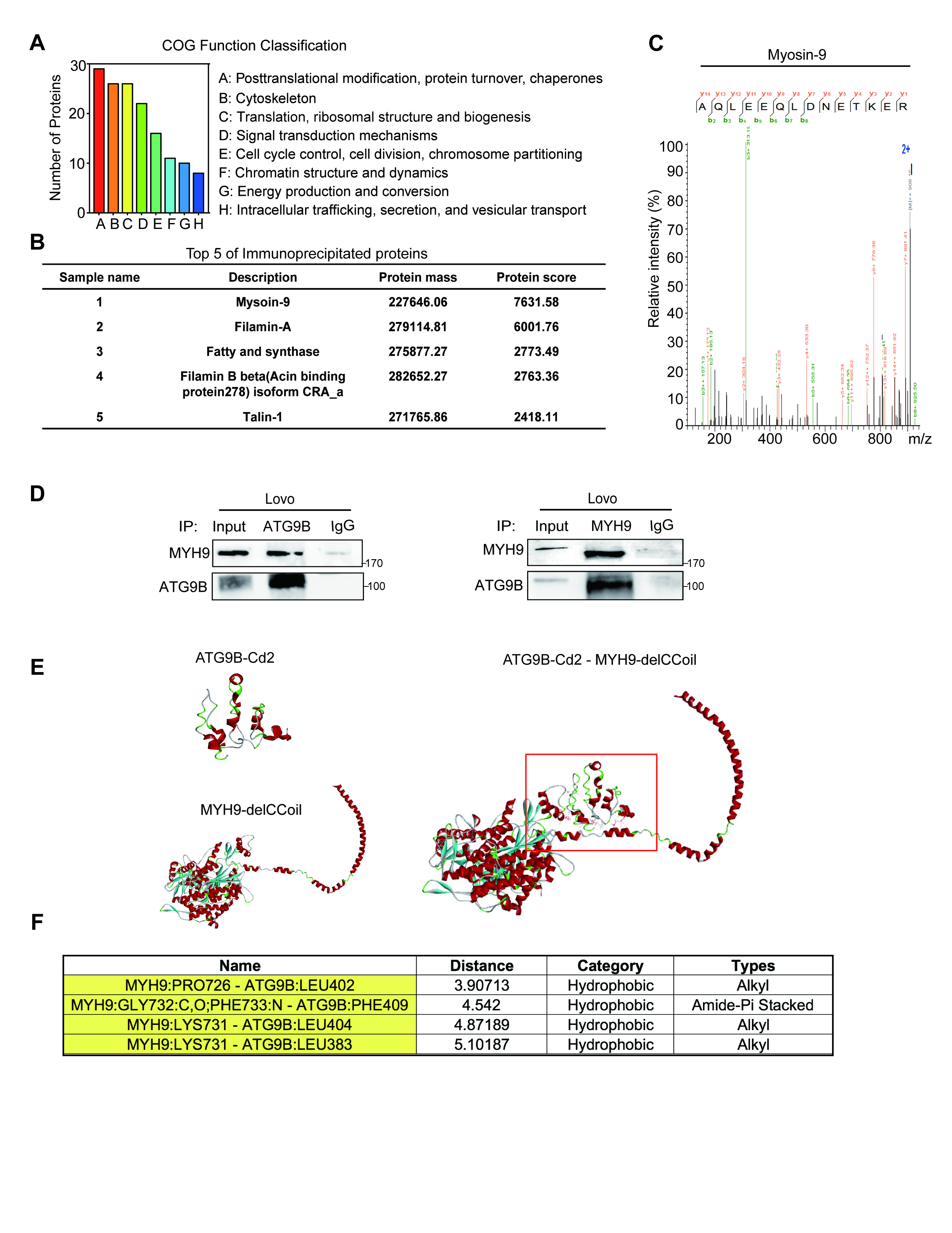

Supplement: Supplementary file 5 — Supplementary Figure 4 [file 41418_2021_813_MOESM5_ESM.tif]

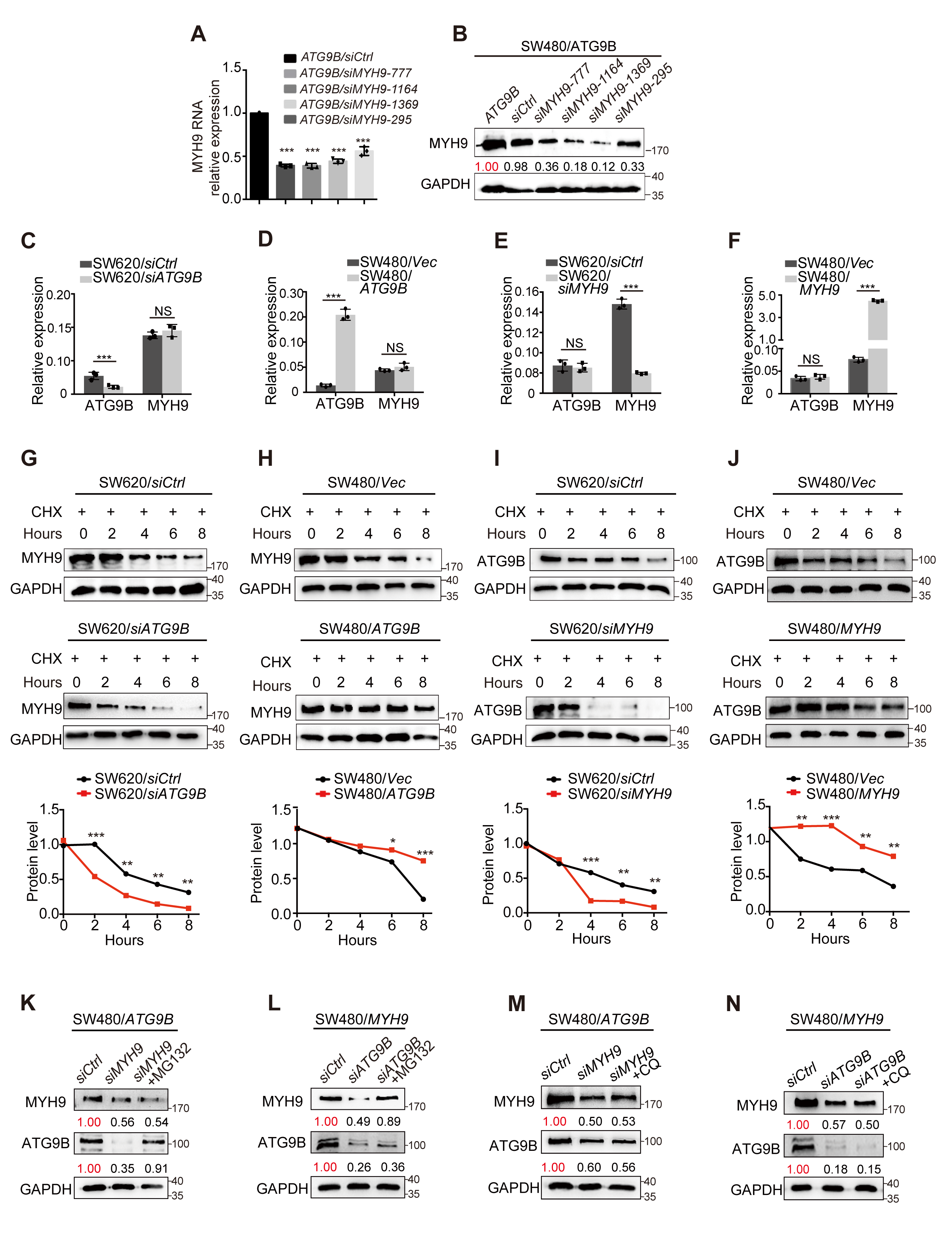

Supplement: Supplementary file 6 — Supplementary Figure 5 [file 41418_2021_813_MOESM6_ESM.tif]

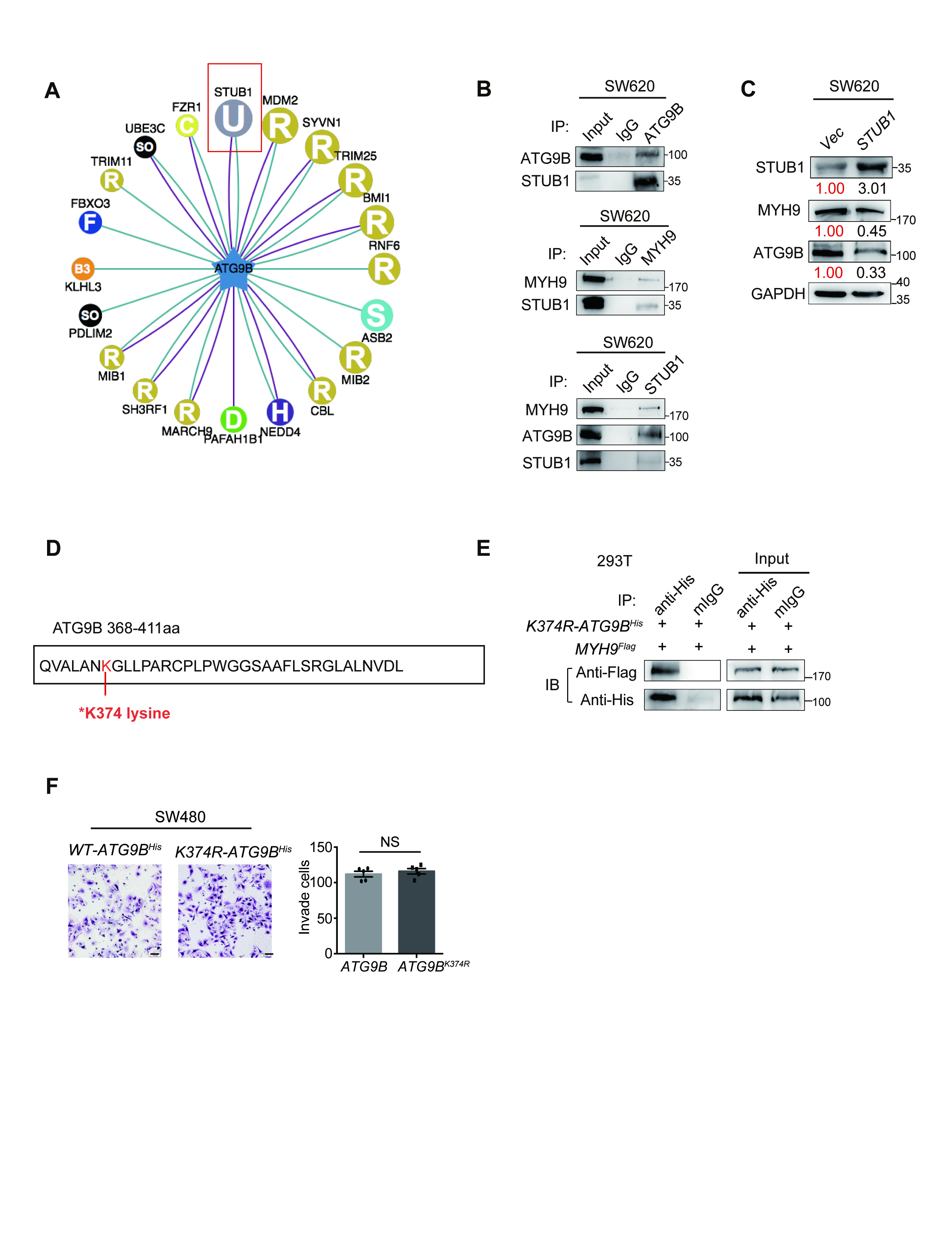

Supplement: Supplementary file 7 — Supplementary Figure 6 [file 41418_2021_813_MOESM7_ESM.tif]

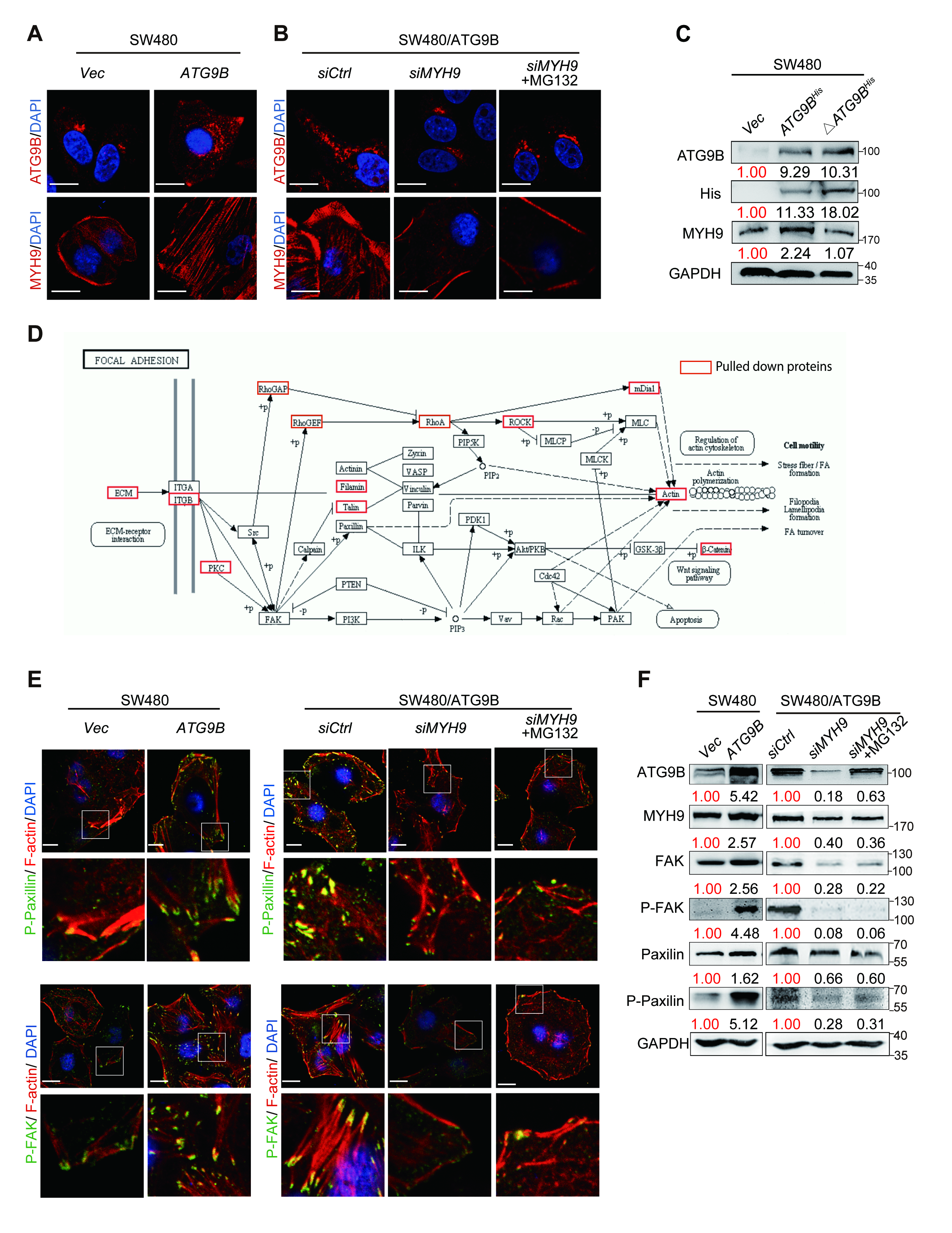

Supplement: Supplementary file 8 — Supplementary Figure 7 [file 41418_2021_813_MOESM8_ESM.tif]

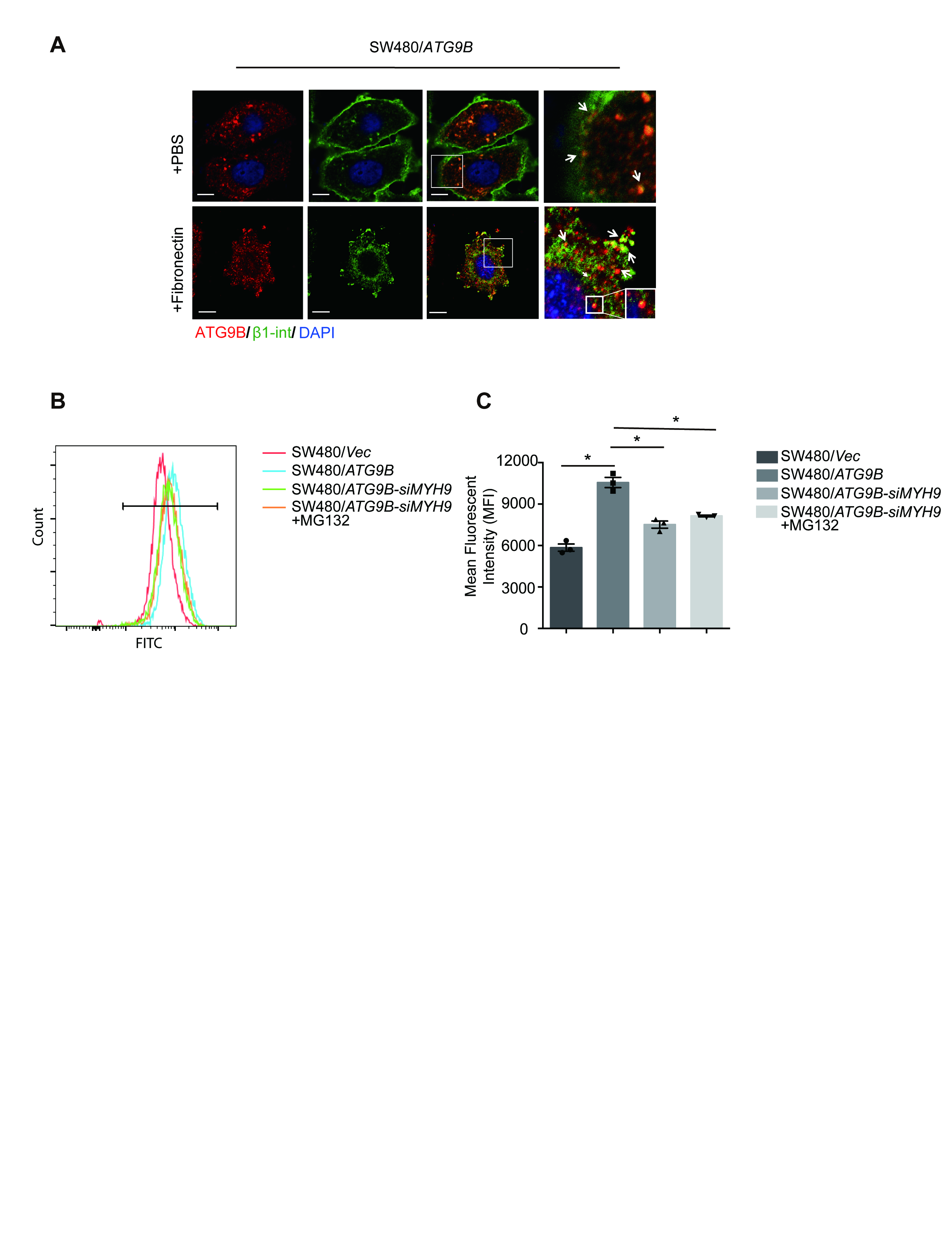

Supplement: Supplementary file 9 — Supplementary Figure 8 [file 41418_2021_813_MOESM9_ESM.tif]

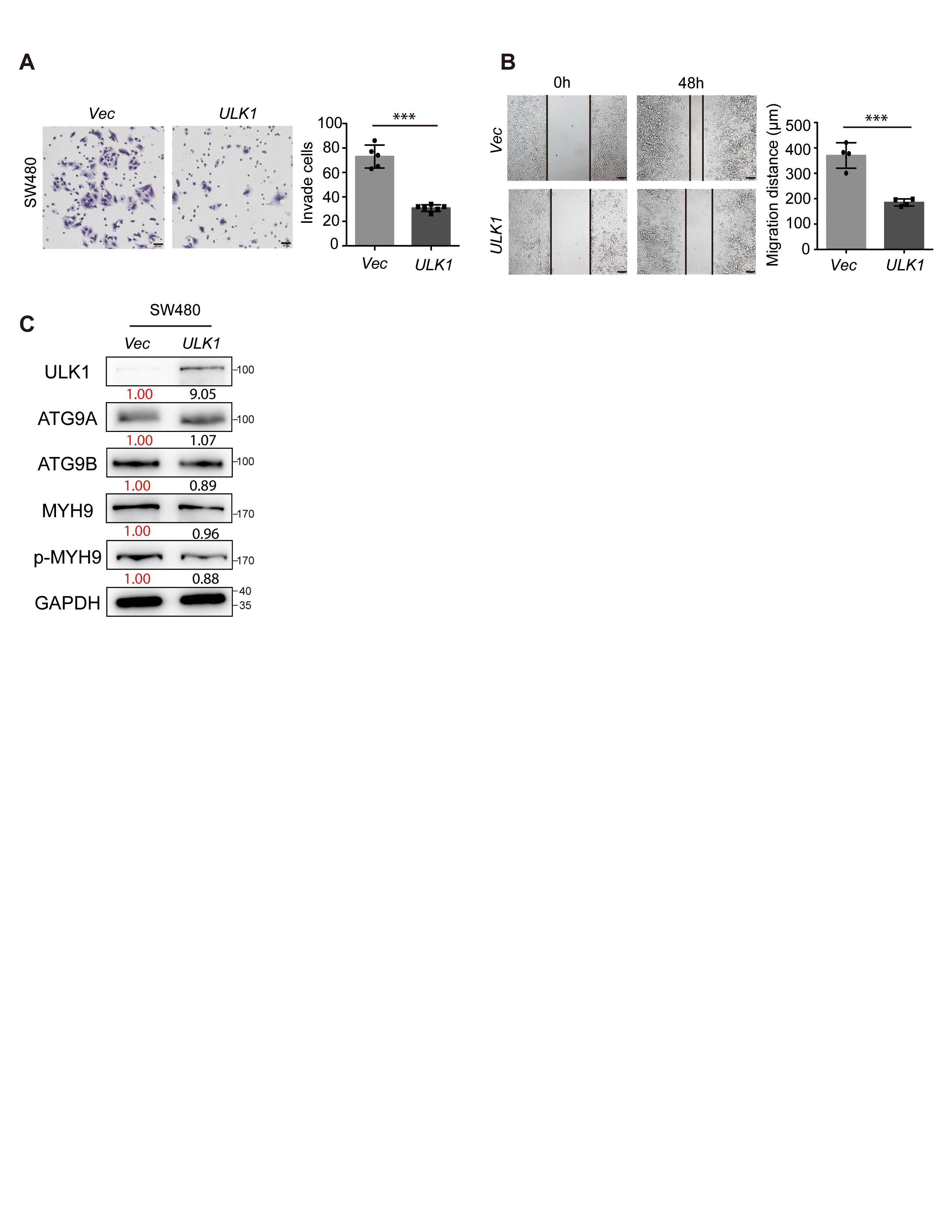

Supplement: Supplementary file 10 — Supplementary Figure 9 [file 41418_2021_813_MOESM10_ESM.tif]
