# Supplementary material for: MYH9-dependent polarization of ATG9B promotes colorectal cancer metastasis by accelerating focal adhesion assembly
Source: Cell Death Differ. 2021 Jun 15;28(12):3251–69. doi: 10.1038/s41418-021-00813-z (PMC8629984; doi:10.1038/s41418-021-00813-z)
Supplement: Supplementary file 11 — Supplementary Table S1 [file 41418_2021_813_MOESM11_ESM.docx]

**Supplementary Table S1**

| **Characteristics** | **Low, n (%)** | **High, n (%)** | **χ2 value** | ***P value*** |
| --- | --- | --- | --- | --- |
| **Frequency (%)** | 38（40.9） | 55（59.1） |  |  |
| **Gender, n (%)** |  |  |  |  |
| Male | 28（34.6） | 44（65.4） | 0.513 | 0.474 |
| Female | 10（47.6） | 11（52.4） |  |  |
| **Age, n (%)** |  |  |  |  |
| ＜50 | 12（30.0） | 28（70.0） | 3.426 | 0.064 |
| ≥50 | 26（49.1） | 27（50.9） |  |  |
| **Tumour size (diameter in cm)** | |  |  |  |
| ＜5 | 20（40.0） | 30（60.0） | 0.033 | 0.856 |
| ≥5 | 18（41.9） | 25（58.1） |  |  |
| **Tumour differentiation** | |  |  |  |
| Good | 14（43.7） | 18（56.3） | 0.326 | 0.849 |
| Moderate | 21（40.4） | 31（59.6） |  |  |
| Poor | 3（33.3） | 6（66.7） |  |  |
| **Depth of tumour invasion** | |  |  |  |
| Mucosa+muscularis | 8（50.0） | 8（50.0） | 0.668 | 0.414 |
| Full-thickness | 30（39.0） | 47（61.0） |  |  |
| **T classification** | |  |  |  |
| T1+T2 | 8 (35.7) | 7 (64.3) | 1.151 | 0.283 |
| T3+T4 | 30 (24.3) | 48 (75.7) |  |  |
| **N classification** | |  |  |  |
| N0 | 29 (59.2) | 20 (40.8) | 14.950 | **0.001** |
| N1 | 5 (16.7) | 25 (83.3) |  |  |
| N2 | 4 (28.6) | 10 (71.4) |  |  |
| **M classification** |  |  |  |  |
| M0 | 35 (46.7) | 40 (53.3) | 5.406 | **0.020** |
| M1 | 3 (16.7) | 15 (83.3) |  |  |
| **Dukes’ classification** |  |  |  |  |
| A | 6 (60.0) | 4 (40.0) | 15.161 | **0.002** |
| B | 24 (58.5) | 17 (41.5) |  |  |
| C | 5 (20.8) | 19 (79.2) |  |  |
| D | 3 (16.7) | 15 (83.3) |  |  |

**Table S1. Correlation of ATG9B expression with clinico-pathological status in 93 cases of patients with CRC.**

Chi-square test was used to analyze the correlation of ATG9B expression with gender, age, tumour size, tumour differentiation, depth of tumour invasion, TNM classification and Duke’s classification. N and M classification and Duke’s classification were significantly correlated with ATG9B expression in CRC patients.
