# Supplementary material for: MYH9-dependent polarization of ATG9B promotes colorectal cancer metastasis by accelerating focal adhesion assembly
Source: Cell Death Differ. 2021 Jun 15;28(12):3251–69. doi: 10.1038/s41418-021-00813-z (PMC8629984; doi:10.1038/s41418-021-00813-z)
Supplement: Supplementary file 12 — Supplementary Table S2 [file 41418_2021_813_MOESM12_ESM.docx]

| **Supplementary Table S2.** | | | | | | | |
| --- | --- | --- | --- | --- | --- | --- | --- |
|  | **Univariate** | | |  | **Multivariate** | | |
| **Variable** | **OR** | **95%CI** | ***P*** |  | **OR** | **95%CI** | ***P*** |
| **ATG9B** | 3.548 | 1.486-8.469 | **0.004** |  | 3.545 | 1.417-8.871 | **0.007** |
| **Gender** | 1.579 | 0.849-2.938 | 0.149 |  | 2.364 | 1.115-5.015 | 0.025 |
| **Age** | 0.698 | 0.309-1.577 | 0.387 |  | 0.476 | 0.194-1.168 | 0.105 |
| **Tumor size** | 1.484 | 0.790-2.786 | 0.220 |  | 2.119 | 1.013-4.433 | 0.046 |
| **Differentiation** | 1.281 | 0.771-2.129 | 0.338 |  | 1.107 | 0.622-1.970 | 0.730 |
| **Invasion** | 2.399 | 0.880-6.536 | 0.087 |  | 0.641 | 0.203-2.023 | 0.448 |
| **Lymph metastasis** | 4.701 | 2.331-9.480 | **<0.001** |  | 0.215 | 0.043-1.084 | 0.063 |
| **Distant metastasis** | 7.076 | 3.603-13.895 | **<0.001** |  | 3.162 | 1.375-7.273 | **0.007** |
| **Dukes’ classification** | 6.003 | 2.834-12.716 | **<0.001** |  | 25.412 | 4.577-141.080 | **<0.001** |

**Table S2:** Univariate and multivariate analyses of individual parameters for correlations with overall survival rate: Cox proportional hazards model. Abbreviations: OR, Odds ratio; CI, Confidence interval.
