# Supplementary material for: MYH9-dependent polarization of ATG9B promotes colorectal cancer metastasis by accelerating focal adhesion assembly
Source: Cell Death Differ. 2021 Jun 15;28(12):3251–69. doi: 10.1038/s41418-021-00813-z (PMC8629984; doi:10.1038/s41418-021-00813-z)
Supplement: Supplementary file 13 — Supplementary Table S3 [file 41418_2021_813_MOESM13_ESM.docx]

**Supplementary Table S3.**

| **Characteristics** | **Non co-high (%)** | **Co-High, n (%)** | **χ2 value** | ***P* value** |
| --- | --- | --- | --- | --- |
| **Frequency (%)** | 31（44.9） | 38（55.1） |  |  |
| **Gender, n (%)** |  |  |  |  |
| **Male** | 19（42.2） | 26（57.8） | 0.383 | 0.536 |
| **Female** | 12（50.0） | 12（50.0） |  |  |
| **Age, n (%)** |  |  |  |  |
| **＜50** | 6（42.9） | 8（57.1） | 0.030 | 0.862 |
| **≥50** | 25（45.5） | 30（54.5） |  |  |
| **T classification** | |  |  |  |
| **T1+T2** | 5（41.7） | 7（58.3） | 0.062 | 0.803 |
| **T3+T4** | 26（45.6） | 31（54.4） |  |  |
| **N classification** |  |  |  |  |
| **N0** | 22（62.9） | 13（37.1） | 9.680 | 0.008 |
| **N1** | 7（30.4） | 16（69.6） |  |  |
| **N2** | 2（18.2） | 9（81.8） |  |  |
| **M classification** |  |  |  |  |
| **M0** | 23（56.1） | 18（43.9） | 5.095 | 0.024 |
| **M1** | 8（28.6） | 20（71.4） |  |  |

**Table S3.** Correlation of ATG9B^high^ MYH9^high^ expression with pathological status in 69 cases of patients with CRC.
