# Supplementary material for: MYH9-dependent polarization of ATG9B promotes colorectal cancer metastasis by accelerating focal adhesion assembly
Source: Cell Death Differ. 2021 Jun 15;28(12):3251–69. doi: 10.1038/s41418-021-00813-z (PMC8629984; doi:10.1038/s41418-021-00813-z)
Supplement: Supplementary file 14 — Supplementary Table S4 [file 41418_2021_813_MOESM14_ESM.docx]

## Supplementary Table S4: List of antibodies and reagents used in the study.

| **Antibody** | **Catalog Number** | **Commercial providers** | **Application** |
| --- | --- | --- | --- |
| ATG9B | ab117591 | Abcam | WB/IP |
| ATG9B | PA5-20998 | ThermoFisher | IF/IHC |
| MYH9 | 11128-1-AP | Proteintech | WB/IP/IF/IHC |
| GAPDH | 60004-1-Ig | Proteintech | WB |
| Tubulin | 66031-1-Ig | Proteintech | WB |
| Beclin1 | #3495 | Cell Signaling Technology | WB |
| ATG12 | #4180 | Cell Signaling Technology | WB |
| ATG7 | #8558 | Cell Signaling Technology | WB |
| ATG5 | #12994 | Cell Signaling Technology | WB |
| P62 | #8025 | Cell Signaling Technology | WB |
| LC3A/B | #12741 | Cell Signaling Technology | WB/IF/IHC |
| His | 66005-1-Ig | Proteintech | WB/IP |
| FLAG | 20543-1-AP | Proteintech | WB/IP |
| FLAG | F2555 | Sigma | WB |
| Ubiquitin | #3936 | Cell Signaling Technology | WB |
| HA | 51064-2-AP | Proteintech | WB/IP |
| Myc | 16286-1-AP | Proteintech | WB/IP |
| STUB1 | 55430-1-AP | Proteintech | WB/IP |
| FAK | 12636-1-AP | Proteintech | WB/IF |
| Tyr397-FAK | #8556 | Cell Signaling Technology | WB/IF |
| Paxillin | 610619 | BD | WB/IF |
| pY118-Paxillin | BS4154 | Bioworld | WB/IF |
| SRC | 11097-1-AP | Proteintech | WB |
| pY418-SRC | ab40660 | Abcam | WB |
| β1-integrin | ab24693 | Abcam | WB/IF/IP |
| Talin-1 | #4021 | Cell Signaling Technology | WB/IP |
| F-actin | #PHDR1 | Cytoskeleton | IF |
