# Supplementary material for: MYH9-dependent polarization of ATG9B promotes colorectal cancer metastasis by accelerating focal adhesion assembly
Source: Cell Death Differ. 2021 Jun 15;28(12):3251–69. doi: 10.1038/s41418-021-00813-z (PMC8629984; doi:10.1038/s41418-021-00813-z)
Supplement: Supplementary file 15 — Supplementary Table S5 [file 41418_2021_813_MOESM15_ESM.docx]

### Supplementary Table S5: List of RNA interference oligo sequences.

| siRNAs | Sense 5’ to 3’ | Anti-sense 5’ to 3’ |
| --- | --- | --- |
| ATG9B-509 | GAUCCCUGAACAGGAUUAUTT | AUAAUCCUGUUCAGGGAUCTT |
| ATG9B-775 | GAUGCGUGGAUUACAAUGUTT | ACAUUGUAAUCCACGCAUCTT |
| ATG9B-1792 | GGUCUUUCAUUCCGGAAGATT | UCUUCCGGAAUGAAAGACCTT |
| MYH9-1164 | CCGUACAACAAAUACCGCUTT | AGCGGUAUUUGUUGUACGGTT |
| MYH9-1365 | GGGUAUCAAUGUGACCGAUTT | AUCGGUCACAUUGAUACCCTT |
| MYH9-295 | GCAAGCUGCCGAUAAGUAU | AUACUUAUCGGCAGCUUGC |
| ATG5-1 | GCGGTTGAGGCTCACTTTA | TAAAGTGAGCCTCAACCGC |
| ATG5-2 | GGGAAGCAGAACCAUACUA | TAGTATGGTTCTGCTTCCC |
| ATG7 | GCCGUGGAAUUGAUGGUAUTT | AUACCAUCAAUUCCACGGCTT |
| β1-Integrin-1 | GGAAAUGGUGUUUGCAAGU | ACUUGCAAACACCAUUUCC |
| β1-integrin-2 | AAUGUAACCAACCGUAGCA | UGCUACGGUUGGUUACAUU |
| FAK | GCAUGUGGCCUGCUAUGGATT | UCCAUAGCAGGCCACAUGCTT |
